# It’s about location, location, location: Absolute and relative stimulus positions in action control

**DOI:** 10.3758/s13414-025-03062-1

**Published:** 2025-04-24

**Authors:** Nicolas D. Münster, Philip Schmalbrock, Christian Frings

**Affiliations:** 1 Department of Psychology, Cognitive Psychology, University of Trier, Universitätsring 15, D-54296 Trier, Germany; 2 Institute for Cognitive & Affective Neuroscience (ICAN), University of Trier, Trier, Germany

**Keywords:** S-R binding, Stimulus feature, Location, Frame of reference

## Abstract

In action control research, stimulus features are assumed to get bound to response features and integrated into an event file. Repetition of any feature leads to retrieval of this event file, causing interference with the current action, depending on whether features repeat or change. It is known that the location of a stimulus works as a feature in these processes. Location is usually operationalized as the absolute position of the stimulus; however, the significance of a particular stimulus location is often only revealed when its position relative to other context stimuli is considered as well. In two experiments (*N*_total_ = 100), we investigated under which conditions which form of location—absolute or relative—is used for binding and retrieval processes. It was shown that solely absolute stimulus location is used when there is a unique absolute target stimulus position for each possible relative target stimulus location. As soon as the target stimulus’ locations can no longer be conclusively defined by its absolute position, relative stimulus locations are used in binding and retrieval processes as well. Results are discussed in terms of prioritization processes and the idea of context-dependent processing of position deviances. Taken together, this reveals a flexible use of location as a feature in action control processes.

**Supplementary Information:**

The online version contains supplementary material available at 10.3758/s13414-025-03062-1.

## Introduction

Imagine reading the time from your analog kitchen clock in order to quench your boiling eggs at the right moment. You direct your gaze to the position on the wall and then determine the position of the hands to read the time. Important for this procedure are the position of the clock in the room and the position of the hands on the clock face. In both cases, the stimulus feature *location*^1^ is involved; however, the frame of reference differs.

At a native (early visual processing) level, the visual information we perceive is processed in retinotopic coordinates (Gardner et al., 2008; Golomb & Kanwisher, 2012; Golomb et al., 2008). That is, its location is processed as where an image lands on the retina. This location is therefore coded in a *retinotopic frame of reference* (Golomb et al., 2010a, 2010b; Golomb, Pulido, et al., 2010). However, since both humans and the world that surrounds us are not static, this information has to be transformed (*remapped*) into a coordinate system that is independent of the gaze and/or the retinal coordinates. For example, when you walk around in the kitchen, you can still recall where in this room the clock is located. This frame of reference is typically called a *spatiotopic frame of reference* (for a review, see Burr & Morrone, 2011). Since location information within this frame of reference has been found to be relevant to behavioral measures (i.e., in a priming task; Tower-Richardi et al., 2016), the present study concentrates on how different, simultaneously applicable spatiotopic frames of reference are processed and used in perception–action integration.

This can be the case, when the location of one stimulus (e.g., the clock on the wall) contains additional location information (e.g., the clock hands)—both information can be relevant, for example, if you want to know the time. Thus, one spatiotopic reference frame is *nested* in another (on the use of spatiotopic frames of reference, see, e.g., Chelazzi et al., 2014; Lee & Shomstein, 2013; Tower-Richardi et al., 2016). Here, we refer to the higher-level, superordinate spatiotopic frame of reference as *world-centered* and to the nested frame of reference as *object-centered*, depending on the relative system that is used to localize a stimulus (e.g., the wall, where the clock is located, or the clock, where the clock hands are located). However, depending on what one's intention is—just locate the clock or read the time—the location information of a stimulus should be accessed differently. Looking *for* the clock needs other location information than *reading* the clock. Thus, location information might be accessed corresponding to the action goal.

How action goals affect our actions and perceptions is investigated in the field of action control research. According to the theory of event coding (TEC; Hommel et al., 2001), the features of all stimuli (S) and responses (R) that occur in a common action episode are bound together (or their corresponding cognitive representations: *feature codes*; Frings et al., 2024) into a so-called event file (Hommel, 2004). Importantly, location has been considered a highly relevant stimulus feature for these processes (e.g., Frings & Moeller, 2010; Hommel, 1998).

Interestingly, re-presenting a stimulus feature bound to a response in a subsequent action episode retrieves the entire event file. This retrieved event file can then affect how we perform in the present episode, based on the number of stimulus–response features that repeat. This leads to different types of situations: If only some features are repeated, it can cause a decrease in performance, known as *partial repetition costs,* because the retrieved event file interferes with the new one being created (Henson et al., 2014). On the other hand, complete changes or complete repetitions do not incur such costs (Hommel et al., 2001; see also Frings et al., 2024, for this line of reasoning). Thus, a current action is affected by what happened directly before it. This effect of previous episodes on present actions is typically referred to as an S-R binding effect.

In action control, location plays a prominent role because, after all, all stimuli we interact with always also carry information about their location. There is consensus that (visual) stimulus features are coded to priority maps, containing not only information about *what* to process but also *where* to direct attention (Bisley & Mirpour, 2019; Zelinsky & Bisley, 2015). While the relevance of location is consensus in the literature, it is unclear how action control processes treat nested location information.

Yet the existing literature on S-R binding effects only looks at stimulus locations in settings that only contain a single, superordinate frame of reference (i.e., the display). However, we know from related fields of research that spatial frames of reference that are nested in superordinate frames of reference can also be used as the primary spatial information (e.g., van Moorselaar & Theeuwes, 2024). Coming back to our example of a wall clock, it contains stimulus features on different within-object positions (i.e., the clock hands), while the clock, in turn, is located in a position in space. Therefore, we were interested in the impact of nested locations on S-R binding.

## The present study

The clock example shows the specific configuration that we aimed to realize in this study: That is, a world-centered frame of reference within which an object is positioned, which in turn provides an object-centered frame of reference. Within the object, the relevant stimuli are located. The question was whether the locations of the relevant stimuli are processed not only in terms of their world-centered frame of reference (as we know from, e.g., Frings & Moeller, 2010; Hommel, 1998, 2022; Singh & Frings, 2020) but also in terms of their object-centered frame of reference. By having participants react to a stimulus that differs in terms of these locations, we aimed to make the processing of stimulus locations in the context of action control visible (through S-R binding effects).

**Fig. 1:**
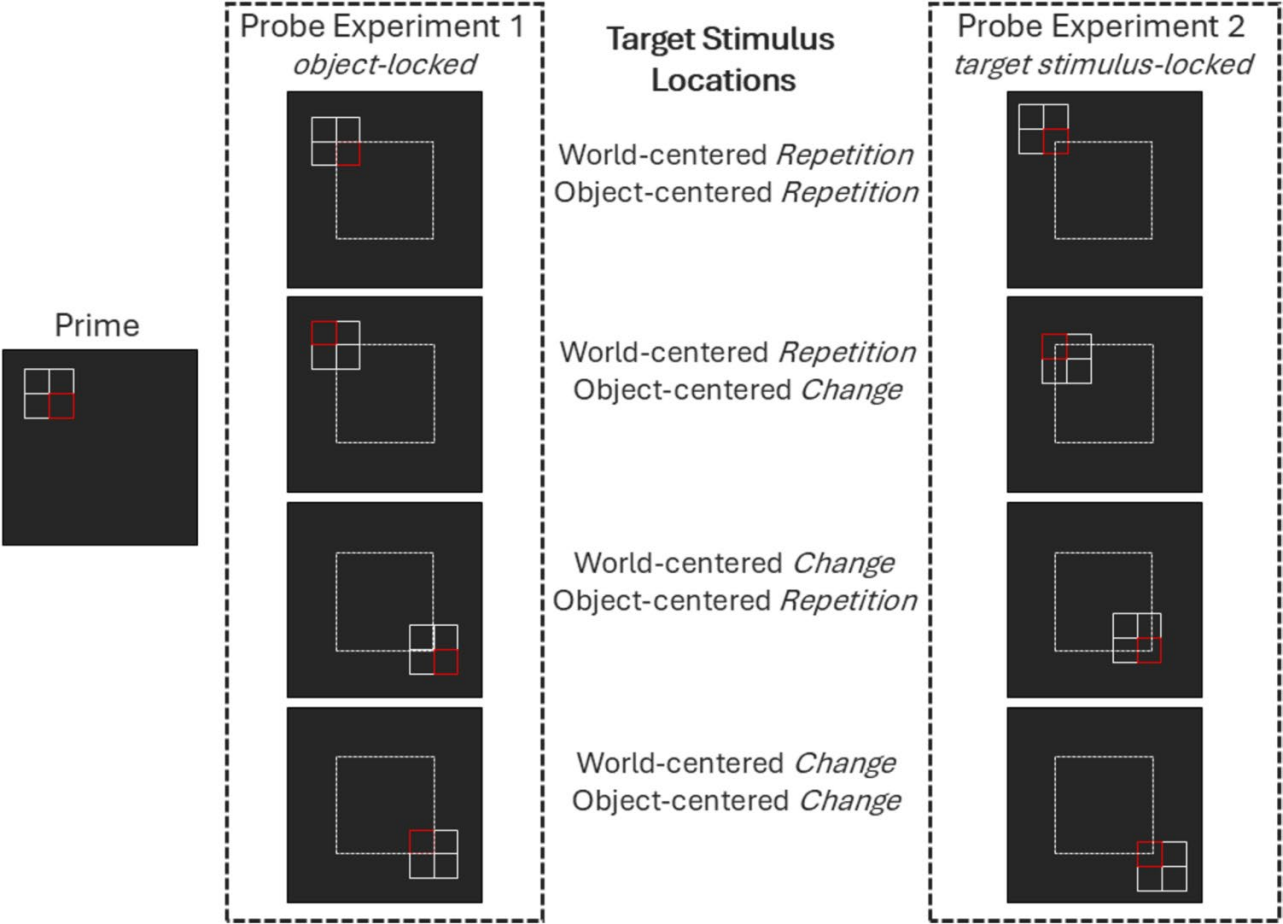
Schematic illustration of target stimulus locations in Experiments 1 and 2. *Note.* Depicted are exemplary prime–probe relations (*repetition* and *change*) of world-centered location and object-centered location in Experiments 1 and 2. The red square marks the target stimulus. The edges of the dotted white square mark the four absolute positions which refer either to the object’s center (Experiment 1) or the target stimulus (Experiment 2). Note that in Experiment 1 the target stimulus was only repeated in terms of its absolute position in trials with world-centered location repetition and object-centered location repetition. In Experiment 2, the target stimulus was repeated in terms of its absolute position in all world-centered location repetition trials, regardless of whether the object-centered location was repeated or changed. The target stimulus could thus appear at 16 different absolute positions in Experiment 1 and at 4 different absolute positions in Experiment 2. The dotted white square did not appear in the experiment. For the actual appearance of the stimuli in the experiments, see Figs. 2 and 3. (Color figure online)

Having two frames of reference, one of which is nested in the other, resulted in two possible experimental operationalizations concerning the absolute and relative position of both the object and the target stimulus. In one variant (Experiment 1), the location of the *object* was chosen so that its center aligned with one of four world-centered locations (in other words, the world-centered location referred to the absolute position of the object, specifically, the object’s center). The target stimulus could be located at one of four positions centered around the object’s center. There were thus 16 (4 × 4) unique absolute positions for the target stimulus depending on the configuration of the object and target stimulus position (see Fig. 1).

**Fig. 2:**
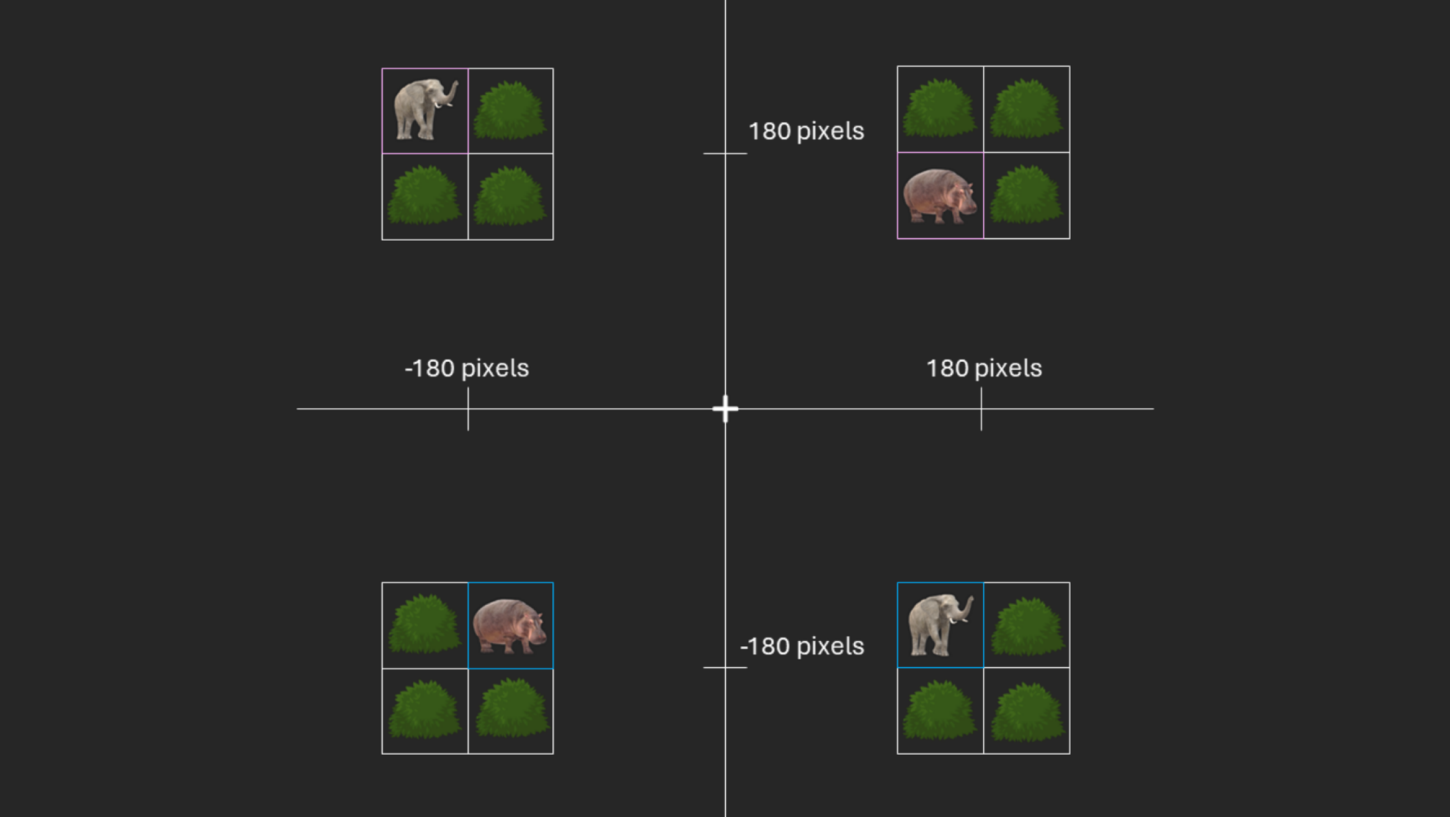
Schematic overview of the possible world-centered (the object’s position on the display) and object-centered locations (the animal’s position within the object) in prime and probe displays in Experiment 1. *Note.* Shown are the four possible world-centered locations of the object (i.e., the quadruplet of squares with bushes/animals), and on each possible location within the object one of the four possible object-centered locations of the target square (containing one of the two different distractor animals, i.e., hippo or elephant. The distractor animal’s identity was random for both prime and probe). The location of the hippo inside the blue square, for example, can be defined in terms of its world-centered location as down left and in terms of its object-centered location as upright. The question was which of those locations would be used in binding and retrieval processes. In the actual prime–probe display, only one of the objects was shown. The axes and axis labels are only drawn to make the world-centered locations clearer and did not appear in the experiment. (Color figure online)

**Fig. 3:**
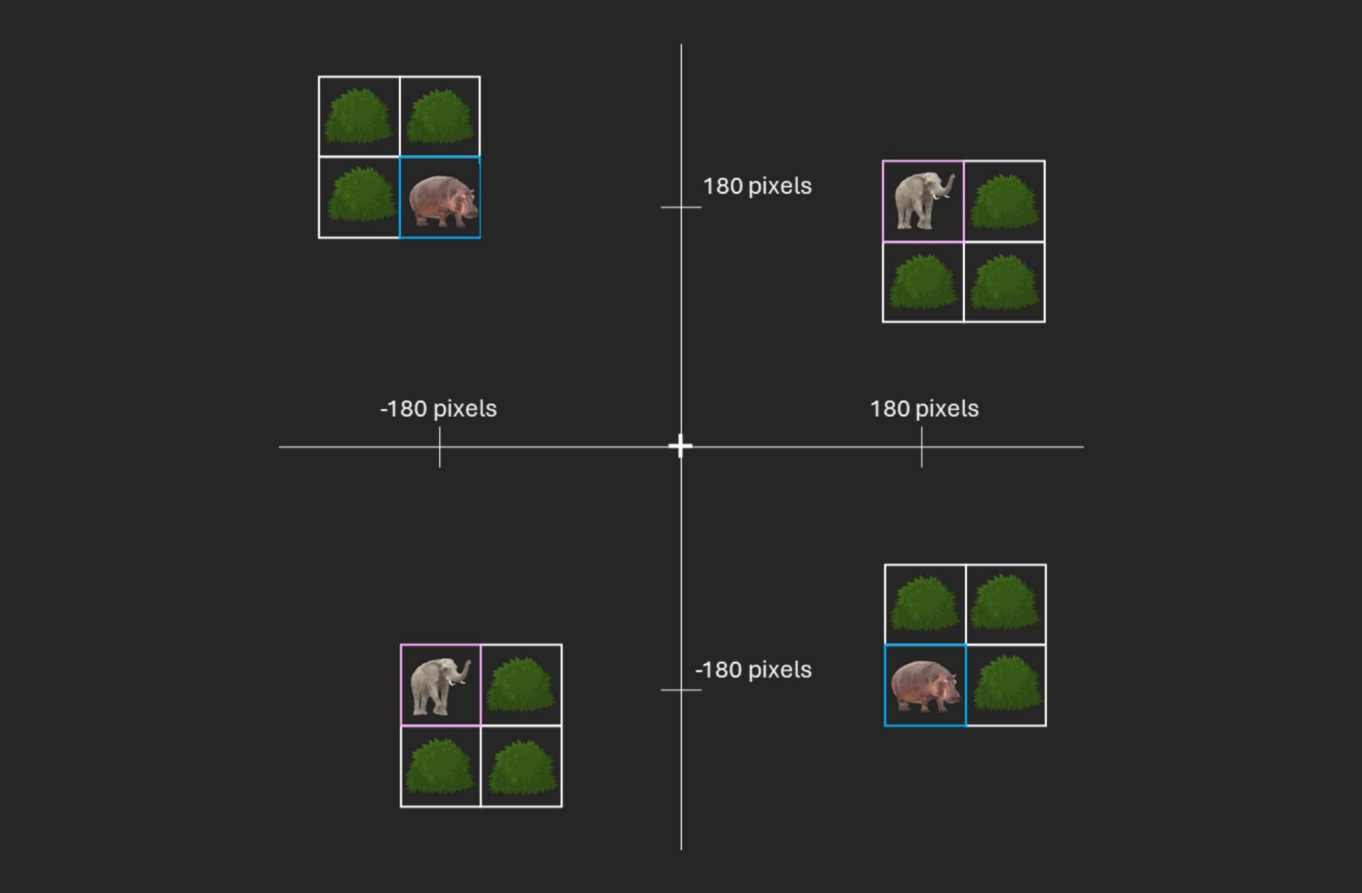
Schematic overview of the possible world-centered and object-centered locations in prime and probe display in Experiment 2. *Note.* Shown are the four possible world-centered locations of the target stimulus, each with one of four possible object arrangements depending on the object-centered condition. In the actual prime/probe display, only one of the objects was shown. The axes and axis labels are only drawn to make the world-centered locations clearer and did not appear in the experiment. (Color figure online)

For differentiation, we call this configuration in Experiment 1 *object-locked*. In the second variant (Experiment 2), the location of the *target stimulus* was chosen so that its center aligned with one of four world-centered locations (in other words, the world-centered location referred to the absolute position of the target stimulus). Consequently, the object could be located as one of four configurations around the target stimulus. This meant that the target stimulus was always at the same position, but the object was situated so that the target stimulus could be in either of its four quadrants (e.g., upper-left corner vs. lower-right corner). There were thus only four absolute positions for the target stimulus, but 16 target–object configurations (with four relative positions of the target stimulus within the object; see Fig. 1). We call this configuration in Experiment 2 *target stimulus-locked*.

To investigate the binding of responses and stimulus locations, we used a typical stimulus–response (S-R) binding paradigm (cf. Frings et al., 2007). In this variant of a sequential priming paradigm, two consecutive displays are presented: a (first) prime and a (second) probe display. Central to our paradigm was a colored frame (the target stimulus). In both prime and probe displays of the prime–probe structure, participants had to respond to the color of this target stimulus, which could repeat or change from prime to probe (response relation). The frame was part of an object (see Fig. 2). The different types of locations were varied as *distractor* stimulus features (i.e., stimulus features that are irrelevant to the participant’s task) of the target stimulus/the colored frame. This means that the target stimulus could change or repeat from prime to probe both in terms of its world-centered location (i.e., the location of the object on the display; world-centered location relation) and in terms of its object-centered location (i.e., the location of the target stimulus within the object; object-centered location relation). Response relation and stimulus feature relations (i.e., world-centered location relation and object-centered location relation) were varied orthogonally. This resulted in trials with complete change or complete repetition of both response relation and stimulus feature relation (which generally leads to performance benefits) and trials with partial repetition: Response was repeated and stimulus feature changed, or vice versa (which generally leads to performance costs). The performance differences resulting from these relations were transformed into S-R binding effects. Since there were two stimulus feature relations (world-centered location relation and object-centered location relation), S-R binding effects_world-centered_ and S-R binding effects_object-centered_ could be computed.

Both variants (Experiments 1 and 2) were of potential interest as they both are realistic but deal with absolute and relative stimulus positions in different ways: In Experiment 1, *only* S-R binding effects for object-centered location in trials with world-centered location change would be indicative of the binding of ‘purely’ relative within-object positions. This is because S-R binding effects for object-centered location in trials with world-centered location repetition were not separable from S-R binding effects for world-centered location, since in both cases it was absolute positions on the display that changed/repeated. In trials with world-centered location change, however, the absolute position of the target stimulus was never repeated. Therefore, only in this condition, S-R binding effects for object-centered location could be clearly attributed to the relative position of the target stimulus within the object. In Experiment 2, *any* S-R binding effects for object-centered location would be clearly attributable to the relative positioning of the target stimulus within the object in both world-centered location change and repetition, since the absolute position of the target stimulus changed/repeated only together with the world-centered location, but never together with the object-centered location.

## Experiment 1: Object-locked

In Experiment 1, we manipulated the world-centered and object-centered locations of distractor stimuli—that is, colored frames that contained semantically loaded pictures of bushes, or, in the case of the target frame, animals (elephant or hippopotamus). Importantly, the bushes and animals only served to give the locations a meaning that was closer to the real world and were not considered in any hypotheses. By using these additional, semantically enriched, and changing animal stimuli, we also aimed to strengthen the expected S-R binding effects for target stimulus locations through processing benefits that arise from changing stimulus material (Schmalbrock et al., 2022).

World-centered location was object-locked, which means that the center of the object was always aligned with the coordinates of the world-centered location. The target stimulus could therefore appear at 16 absolute positions on the display (as a function of world-centered location and object-centered location).

### Method

#### Transparency and openness

We report how we determined our sample size, any data exclusions, all manipulations, and all measures in this study. No Experiment was preregistered (because no nonspeculative directed hypotheses could be made based on the existing literature). This study was done in accordance with the ethical guidelines declared by the ethics committee of Trier University. The ethics committee of Trier University declared all simple behavioral studies in accordance with their guidelines exempt from any further examinations by the committee.

#### Participants

The sample size was calculated according to previous studies investigating S-R binding effects for irrelevant (distractor) stimulus feature dimensions (i.e., stimulus features that change systematically but are not response-relevant; e.g., in Frings et al., 2007; Frings et al., 2013; Singh & Frings, 2020). According to these studies, we expected to find at least a medium-sized effect (*d*_*z*_ ~ 0.40)^2^ for the irrelevant dimension x response repetition interaction (i.e., the binding effect). Thus, we planned to run at least *n* = 52 participants, leading to a power of 1 − β = 0.80 (assuming an α = 0.05, two-tailed; G*Power 3.1.9.7, Faul et al., 2007). Accordingly, 53 students (to account for potential outliers) from Trier University were recruited via the university’s online recruiting system in the spring of 2024. Two participants were excluded because they were heavy outliers (following the classification by Tukey, 1977, pp. 39–43).^3^ Fifty-one participants remained (40 women, 10 men, one other; 50 right-handed, one ambidextrous; *M* = 22.84 years, range: 18–43). Students received credits for their study participation.

#### Design

Four within-participant factors were varied: response relation (response repetition vs. change), world-centered location relation (world-centered repetition vs. change), object-centered location relation (object-centered repetition vs. change), and animal identity relation (identity repetition vs. change).^4^

#### Apparatus and stimuli

The experiment was programmed in PsychoPy (Version 2023.2.1, Peirce et al., 2019) and run online via Pavlovia. Two displays (prime and probe) were presented in each trial. All stimuli were presented on a dark grey (RGB_255:_ 38, 38, 38) background. In the prime display, a quadruplet containing the pictures of three bushes and one animal (56 × 56 pixels, taken from Paint 3D, Microsoft Corporation, arranged in a square) was presented in one of four possible locations (with the center of the quadruplet positioned 180 pixels up/down and right/left from zero; see Fig. 2).

The pictures were presented in squares (60 × 60 pixels), which together formed the object (a large ‘box’, 120 × 120 pixels). The border color of the squares depended on the response condition and the contained picture: The squares containing pictures of a bush always had a white border color (RGB_255:_ 255, 255, 255), the square containing the animal had a blue (RGB_255:_ 0, 176, 255) or pink (RGB_255:_ 255, 176, 255) border color, depending on the response condition. The probe display showed the same configuration as the prime display with the world-centered location, the object-centered location, and the border color depending on the prime display and the response condition.

#### Procedure

Participants were presented with written instructions (in German) of the experimental procedure on the screen. To standardize the online setting as much as possible, they were instructed to orient their keyboard so that the *B* key was in line with the midline of their body. Participants were then instructed to place the left index finger on the *D* key and the right index finger on the *K* key. Another display informed the participants that the task should be completed as quickly and correctly as possible. Finally, the two animals were introduced to the participants by name ('Niko'the hippopotamus, and'Emil'the elephant) to give the participants the feeling that the animal distractors were two individual animals.^5^ Before the experimental block started, participants had to run through eight training trials. During this training, there was written feedback (appearing for 1,000 ms, ‘correct’ or ‘incorrect’, respectively) after each given response. By contrast, in the experimental block, only incorrect answers or answers given too late were commented on. The maximum time for the response in both prime and probe displays was 2,000 ms. In each trial of the experimental block, participants had to respond twice: With the onset of the prime display, participants had to perform a response depending on the color of the frame by pressing either the D-key (for a blue frame) or the K-key (for a pink frame).

**Fig. 4:**
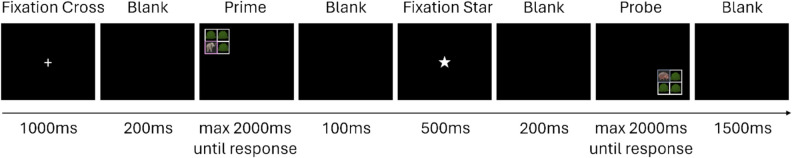
Depiction of an exemplary trial sequence in Experiment 1. *Note.* Shown is one of eight possible trial combinations (as a function of response/world-centered location/object-centered location/animal repetition/change): response change, world-centered location change, object-centered location change, and animal change. Stimuli are not drawn to scale

The experimental block consisted of 512 trials. After every 20th trial, there was a break (participants could continue with the experiment by pressing the space bar). A single trial consisted of the following display sequence (see Fig. 4): A fixation cross was presented in the center of the screen for 1,000 ms, followed by a blank screen (200 ms). Then the prime display was shown for a maximum of 2000 ms (the response ended the display; if no response was given during this time, a message appeared reminding the participant to respond more quickly). Here, the world-centered location of the object, as well as the object-centered location of the colored frame as well as the color of the frame depended on the trial condition, so each condition was displayed 32 times. After an interstimulus interval of 100 ms, a fixation star^6^ appeared for 500 ms. After another blank screen (200 ms) the probe display was shown for a maximum of 2,000 ms, during which the participants had to identify the color of the frame again. The response ended the presentation of the probe stimulus, and the sequence was terminated by a 1,500 ms blank display. If the participants reacted incorrectly, a corresponding message appeared. Each condition was displayed 32 times.

The response relation (response repetition vs. change), the world-centered location relation (world-centered repetition vs. change), the object-centered location relation (object-centered repetition vs. change), and the animal identity relation (identity repetition vs. change) were varied orthogonally. Therefore, neither the world-centered location, the object-centered location, the color of the frame, the animal, nor the response in the prime display predicted the world-centered location, the object-centered location, the color of the frame, the animal, or the response in the probe display. That resulted in various prime–probe relations: On trials with response repetition, the same response that was required in the prime was also required in the probe. Conversely, on trials with response change, the required response differed from prime to probe. On trials with animal identity repetition, the same animal was shown in both prime and probe display; on trials with animal identity change, the animal in the probe display differed from the animal in the prime display. Furthermore, the world-centered location of the object could repeat (world-centered repetition) or change (world-centered change) from prime to probe, as could the object-centered location within the object (object-centered repetition vs. change). In world-centered location change trials, the location of the probe object varied equally on one of the three remaining positions. In other words, with a 50% probability, the prime location was repeated in the probe, and with a 1/6 probability each (together also 50%), the probe location was one of the remaining three other locations (which one was drawn at random). The same applied to object-centered location change trials.

### Results

Data processing and analysis were performed with R (Version 4.2.2; R Core Team, 2019) using a mixed-effects analysis of variance (ANOVA) with Type III sums of squares (‘ezAnova’ function of *ez*; Lawrence, 2016). S-R binding effects were calculated using this formula: [Performance _Response-Repetition/Feature-Change Trials—_Performance _Response-Repetition/Feature-Repetition Trials_] − [Performance _Response-Change/Feature-Change Trials_ − Performance _Response-Change/Feature-Repetition Trials_] (taken from Frings et al., 2007), which can be understood as the benefit of stimulus feature repetition in response-repetition trials minus the cost of stimulus feature repetition in response-change trials.^7^ This is just one way of combining the different trial types resulting from the event file logic (i.e., full repetition, full change, and partial repetition trials) into a single value. Statistically, the significance test for the two-way interaction between response relation and stimulus feature relation in the ANOVA corresponds to the *t* test of the S-R binding effects against zero.

In the present study, stimulus feature relation could either refer to the prime–probe relation of the world-centered location of the target stimulus (repetition vs. change, S-R binding effect_world-centered_) or the prime–probe relation of the object-centered location of the target stimulus within the object (repetition vs. change, S-R binding effect_object-centered_). S-R binding effects were compared with exploratory post hoc *t* tests.

To better evaluate (nonsignificant) effects, we provide Bayes values for *t* tests in addition to frequentist *p* values. Because our sample size planning is based on frequentist statistics, the Bayesian values should only be understood as exploratory interpretation aids. However, it is highlighted in the text if the interpretations associated with the Bayes values do not correspond to the *p* values (interpretations of Bayes factors [BFs] according to Wagenmakers et al., 2011).

Bayesian *t* tests were performed with a standard Cauchy prior with *r* scale = √2/2 (Rouder et al., 2009). Bayes factors (BF_01_) quantify the evidence in favor of the null hypothesis relative to the evidence in favor of the alternative hypothesis. Values between 1 and 3 indicate anecdotal evidence in favor of the null hypothesis, and values > 3 indicate substantial to strong evidence for the null hypothesis. Vice versa, values from 0.33 to 1 indicate anecdotal evidence for the alternative hypothesis, and values < 0.33 indicate substantial to strong evidence for the alternative hypothesis (cf. Wagenmaker et al., 2011).

### Data processing

Trials without given responses (in either prime or probe) were excluded from all analyses (1.2% of trials), as were trials with wrong responses in the prime (8.3% of trials). Also, 4.3% of all trials had correct responses in the prime and wrong responses in the probe, and these trials were used in the error rate analyses. After excluding 3.4% of trials with probe RTs below 200 ms and more than 1.5 interquartile ranges above the third quartile of each subject’s RT distribution (cf. Tukey, 1977), 82,9% of trials remained for reaction time analyses.

### Reaction times

We analyzed whether the S-R binding effects for world-centered location, which we expected from the literature, could be found, as well as S-R binding effects for object-centered location. We then looked at the interaction between both types of locations. Based on this, and most importantly, we tested whether S-R binding effects for object-centered location in trials with world-centered location change could be found.

Therefore, a 2 (response relation: repetition vs. change) × 2 (world-centered location relation: repetition vs. change) × 2 (object-centered location relation: repetition vs. change) ANOVA on probe RTs was conducted. The factor animal identity relation was not included in this ANOVA, as it was not relevant to our hypotheses (for the exploratory ANOVA output including animal identity relation, see Appendix 1D).

A significant two-way interaction between response relation and world-centered location relation was found, indicating S-R binding effects for response and world-centered location (i.e., the overall S-R binding effect_world-centered_): *F*(1, 50) = 105.78, *p* < 0.001, ${{\upeta }_{G}}^{2}$ = 0.01, ${{\upeta }_{p}}^{2}$ = 0.68. This corresponds to the S-R binding effects of absolute stimulus locations that have been observed before.

Another significant two-way interaction was found between response relation and object-centered location relation, indicating S-R binding effects for response and object-centered location (i.e., the overall S-R binding effect_object-centered_): *F*(1, 50) = 33.45, *p* < 0.001, ${{\upeta }_{G}}^{2}$ < 0.01, ${{\upeta }_{p}}^{2}$ = 0.40.

The two-way interaction between world-centered location relation and object-centered location relation reached significance as well, *F*(1, 50) = 20.32, *p* < 0.001, ${{\upeta }_{G}}^{2}$ < 0.01, ${{\upeta }_{p}}^{2}$ = 0.29, and, also importantly, the three-way interaction between response relation, world-centered location relation, and object-centered location relation: *F*(1, 50) = 24.81, *p* < 0.001, ${{\upeta }_{G}}^{2}$ < 0.01, ${{\upeta }_{p}}^{2}$ = 0.33. This indicates that repetition and change of one type of location influenced how repetition and change of the other type of location contributed to S-R binding effects. In other words, the overall S-R binding effect_world-centered_ was modulated by whether the object-centered location was repeated or changed, and the overall S-R binding effect_object-centered_ was modulated by whether the world-centered location was repeated or changed.

**Fig. 5:**
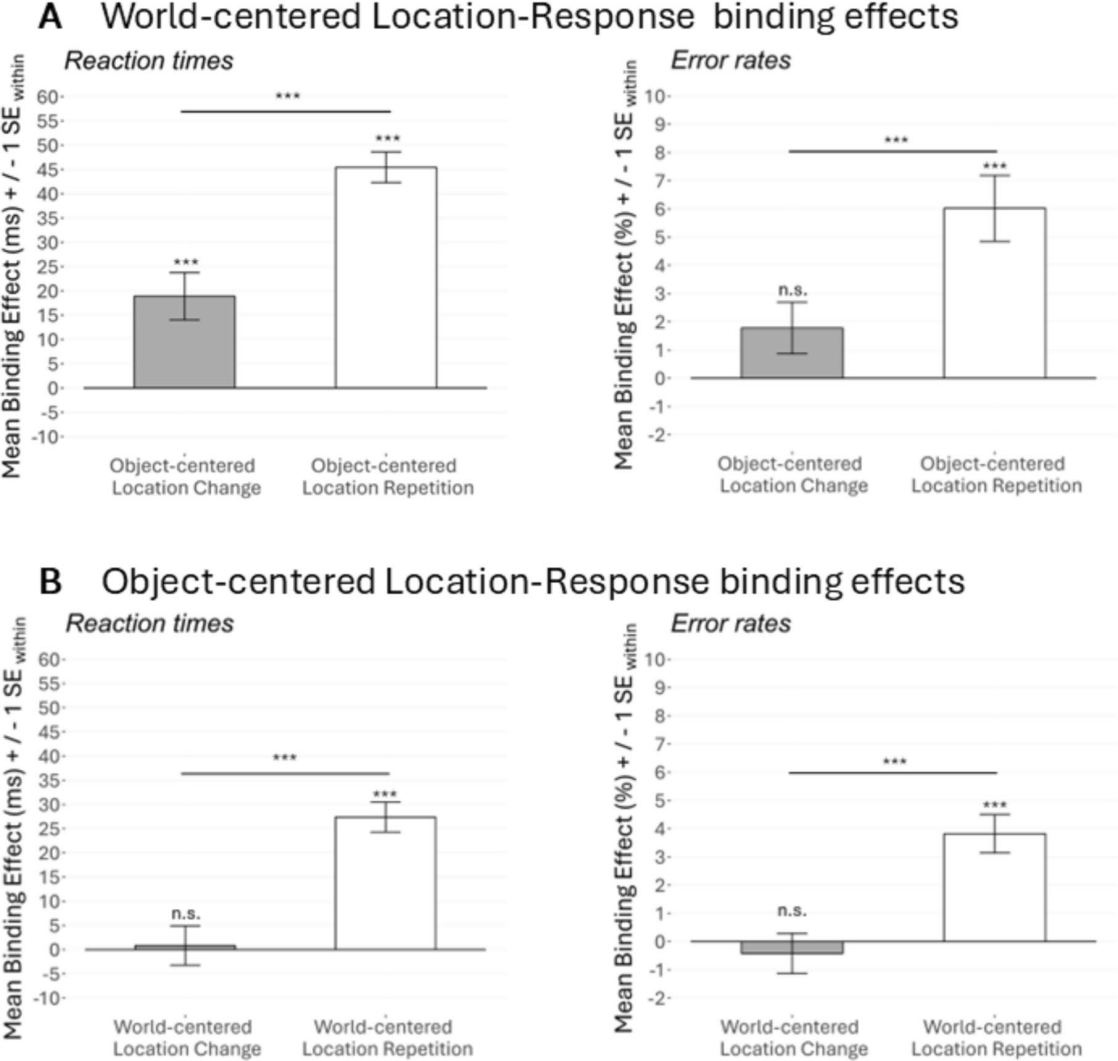
S-R binding effects in Experiment 1. *Note*. Panel **A** shows S-R binding effects (reaction times and error rates) for world-centered location in trials with object-centered location change and repetition. Panel **B** shows S-R binding effects for object-centered location in trials with world-centered location change and repetition. See Appendices 1B & 1 F for a depiction of all single data points that contribute to the binding effects. n.s. = *p* > 0.050, *p < 0.050, **p < 0.010, ***p < 0.001

To take a closer look at this three-way interaction, we exploratively conducted post hoc *t* tests of the calculated S-R binding effects (calculated with the formula described above). These post hoc *t* tests first revealed a significant difference for binding effect_world-centered_ between the two stages of object-centered location relation (i.e., repetition or change): two-tailed* t(50) = 4.98, p* < 0.001, *d*_z_ = 0.70, BF_01_ < 0.01 (see Fig. 5A).

When testing against zero, the binding effect_world-centered_ reached significance in both stages of object-centered location relation: In trials with object-centered location repetition (*M* = 45 ms, *SD* = 23 ms; two-tailed), *t(50) = 14.39, p* < 0.001, *d*_z_ = 2.01, BF_01_ < 0.01, and in trials with object-centered location change (*M* = 19 ms, *SD* = 35 ms; two-tailed), *t(50) = 3.87, p* < 0.001, *d*_z_ = 0.54, BF_01_ = 0.01. This indicates that the S-R binding effect for world-centered location occurred in trials with both repetition and change of object-centered location. However, the strength of the S-R binding effect for world-centered location was modulated by whether the object-centered location was repeated or changed.

Important for our research question, the binding effect_object-centered_ only reached significance in trials with world-centered location repetition (*M* = 27 ms, *SD* = 22 ms; against zero: two-tailed), *t(50) = 8.82, p* < 0.001, *d*_z_ = 1.24, BF_01_ < 0.01, but not in trials with world-centered location change (*M* = 1 ms, *SD* = 29 ms; against zero: two-tailed), *t(50) = 0.21, p* = 0.837, *d*_z_ = 0.03, BF_01_ = 6.43, against each other: two-tailed,* t(50) = 4.98, p* < 0.001, *d*_z_ = 0.70, BF_01_ < 0.01 (see Fig. 5B). This indicates that the binding effect_object-centered_ only emerged when the world-centered location was repeated, but not when the world-centered location was changed from prime to probe.

### Error rates

Following the analyses of reaction times, we conducted a 2 (response relation: repetition vs. change) × 2 (world-centered location relation: repetition vs. change) × 2 (object-centered location relation: repetition vs. change) ANOVA on probe error rates. As in reaction times, the factor animal identity relation was not included in this ANOVA (for the exploratory ANOVA output including animal identity relation, see Appendix 1H).

A significant two-way interaction between response relation and world-centered location relation could be found, supporting the S-R binding effects for response and world-centered location that were also found in reaction times (overall binding effect_world-centered_): *F*(1, 50) = 19.52, *p* < 0.001, ${{\upeta }_{G}}^{2}$ = 0.05, ${{\upeta }_{p}}^{2}$ = 0.28.

Also as in reaction times, the two-way interaction between response relation and object-centered location relation (overall binding effect_object-centered_) reached significance: *F*(1, 50) = 17.56, *p* < 0.001, ${{\upeta }_{G}}^{2}$ = 0.01, ${{\upeta }_{p}}^{2}$ = 0.26.

Unlike in the reaction times, no two-way interaction between world-centered location relation and object-centered location relation could be found, *F*(1, 50) = 0.82, *p* = 0.371, ${{\upeta }_{G}}^{2}$ < 0.01, ${{\upeta }_{p}}^{2}$ = 0.02. However, the three-way interaction between response relation, world-centered location relation, and object-centered location relation reached significance: *F*(1, 50) = 14.02, *p* < 0.001, ${{\upeta }_{G}}^{2}$ = 0.02, ${{\upeta }_{p}}^{2}$ = 0.22. This supports the finding observed in reaction times: The prime–probe relations of both types of locations influenced each other in the strength of their respective S-R binding effects.

Therefore, as in reaction times, exploratory post hoc *t* tests of calculated S-R binding effects revealed a significant difference for binding effect_world-centered_ between repetition and change of the object-centered location relation as well as a significant difference for binding effect_object-centered_ between repetition and change of the world-centered location relation (see Fig. 4A and B). Both these differences: two-tailed,* t(50) = 3.74, p* < 0.001, *d*_z_ = 0.52, BF_01_ = 0.02 (note that *t* tests for both variants yield the same result since they correspond to the three-way interaction of the ANOVA). The binding effect_world-centered_ in both stages of object-centered location relation was tested against zero: In trials with object-centered location repetition, the binding effect_world-centered_ reached significance (*M* = 5.02%, *SD* = 8.35%; two-tailed), *t(50) = 5.15, p* < 0.001, *d*_z_ = 0.72, BF_01_ < 0.01, but not (frequentist) respectively was anecdotal (Bayesian) in trials with object-centered location change: *M* = 1.78%, *SD* = 6.52%; two-tailed, *t(50) = 1.95, p* = 0.057, *d*_z_ = 0.27, BF_01_ = 1.14. This supports the pattern found in the reaction times.

Importantly, and also comparable to reaction times, the binding effect_object-centered_ only reached significance in trials with world-centered location repetition (*M* = 3.82%, *SD* = 4.85%; two-tailed), *t(50) = 5.63, p* < 0.001, *d*_z_ = 0.79, BF_01_ < 0.01, but not in trials with world-centered location change (*M* = − 0.42%, *SD* = 5.09%; two-tailed), *t(50) = 0.59, p* = 0.557, *d*_z_ = 0.08, BF_01_ = 5.56. This underlines that a binding effect for object-centered location only then emerged when the world-centered location was repeated, but not when the world-centered location was changed from prime to probe.

## Discussion

In Experiment 1, we replicated previous findings, after which responses and world-centered locations were bound. Yet the binding effect was smaller (but still significant), when the object-centered location was changed compared with trials in which the object-centered location was repeated.

Importantly, however, the object-centered location only caused S-R binding effects when the world-centered location was repeated. In trials with world-centered location change, in turn, no S-R binding effects for object-centered location were observed. This means that the S-R binding effect, which could be detected for the relative position of the target stimulus within the object, can also be explained by the absolute target stimulus position. It is therefore possible that only the changes and repetitions of the *absolute* stimulus positions caused the observed S-R binding effects for object-centered location. This would also be consistent with the lack of S-R binding effects in the ‘purely’ relative condition: Here, due to world-centered location change, the absolute target stimulus position was never repeated.

However, the possibility cannot be ruled out that only if the object’s location in the superordinate, world-centered frame of reference is repeated, the location within the object (i.e., within the nested, object-centered frame of reference) is processed and used for binding and retrieval. Also, the fact that all 16 possible target stimulus locations had a unique absolute position could have led to the relative positions being ignored, as they did not provide any additional information.

To address the question of whether the usage of relative stimulus positions in binding and retrieval processes depends on the uniqueness of the stimulus’ absolute position, we conducted Experiment 2. Here, we changed the way the object and the target stimulus were positioned on the display.

## Experiment 2: Target stimulus-locked

In Experiment 2, objects were target stimulus-locked. That means that a relative position change of the target stimulus within the object changed the absolute position of the object, but not the absolute position of the target stimulus itself. By doing so, the absolute position of the target stimulus only changed with the world-centered location, but not with the object-centered location. The target stimulus could therefore only appear at four absolute positions on the display, but at each of the absolute positions in four different relative constellations (so, again, a total of 16 locations as a function of four world-centered and four object-centered locations).

### Method

#### Transparency and openness

Transparency and openness was identical to Experiment 1.

#### Participants

The sample size was calculated analogously to Experiment 1—that is, we planned for S-R binding effects (for the response-irrelevant stimulus features) with an estimated effect size of *d*_z_ = 0.4 according to previous findings of S-R binding effects for irrelevant stimulus features (e.g., in Frings et al., 2007; Frings et al., 2013; Singh & Frings, 2020). Thus, we again planned to run *n* = 52 participants. Accordingly, another 53 participants (to account for potential outliers) from Trier University were recruited via the university’s online recruiting system in the spring of 2024. Four participants were excluded because they were heavy outliers (following the classification by Tukey, 1977, pp. 39–43).^8^ Forty-nine participants remained (40 women, nine men; 44 right-handed, five left-handed; *M* = 22.12 years, range: 18–31). Students received credits for their study participation.

#### Design

The same four within-participant factors were varied as in Experiment 1: response relation (response repetition vs. change), world-centered location relation (world-centered repetition vs. change), object-centered location relation (object-centered repetition vs. change), and animal identity relation (identity repetition vs. change).^9^

#### Apparatus and stimuli

Apparatus and stimuli were identical to Experiment 1 with the exception that the target stimulus only appeared at four absolute positions (180 pixels in each dimension), depending on the world-centered location and independent of the object-centered location (see Fig. 5). The remaining three squares (building the object together with the target stimulus) were therefore placed *around* the target stimulus, depending on the object-centered location.

#### Procedure

The procedure and trial sequence were identical to Experiment 1.

### Results

Data analyses were performed analogously to Experiment 1. Again, Bayes values for *t* tests should only be understood as exploratory interpretation aids.

#### Data processing

Trials with not given responses (in either prime or probe, 3.3% of trials) as well as trials with wrong responses in the prime (7.5% of trials) were excluded from all. 4.5% of all trials with correct responses in the prime and wrong responses in the probe were used in the error rate analyses. Another 4.6% of trials with probe RTs below 200 ms and more than 1.5 interquartile ranges above the third quartile of each subject’s RT distribution (cf. Tukey, 1977) were excluded, so that 80.9% of trials remained for reaction time analyses.

#### Reaction times

As in Experiment 1, we analyzed whether S-R binding effects for both world-centered location and object-centered location could be found. We also checked whether both types of locations would interact with each other. Based on that, we were particularly interested in S-R binding effects for object-centered location in trials with world-centered location change, since in this condition the absolute location of the target stimulus was identical between prime and probe displays and therefore provided the ‘cleanest’ opportunity to detect S-R binding effects that were uniquely driven by the relative position of the target stimulus within the object.

A 2 (response relation: repetition vs. change) × 2 (world-centered location relation: repetition vs. change) × 2 (object-centered location relation: repetition vs. change) ANOVA on probe RTs was conducted. As in Experiment 1, the factor animal identity relation was not included in this ANOVA, as it was not relevant to our hypotheses (for the ANOVA output including animal identity relation, see Appendix 2D).

First, a significant two-way interaction between response relation and world-centered location relation was found, indicating S-R binding effects for response and world-centered location (overall S-R binding effect_world-centered_), *F*(1, 48) = 78.45, *p* = 0.001, ${{\upeta }_{G}}^{2}$ = 0.01, ${{\upeta }_{p}}^{2}$ = 0.62.

Importantly, a significant two-way interaction was found between response relation and object-centered location relation, indicating S-R binding effects for response and object-centered location (overall S-R binding effect_object-centered_), *F*(1, 48) = 50.28, *p* < 0.001, ${{\upeta }_{G}}^{2}$ < 0.01, ${{\upeta }_{p}}^{2}$ = 0.51.

**Fig. 6:**
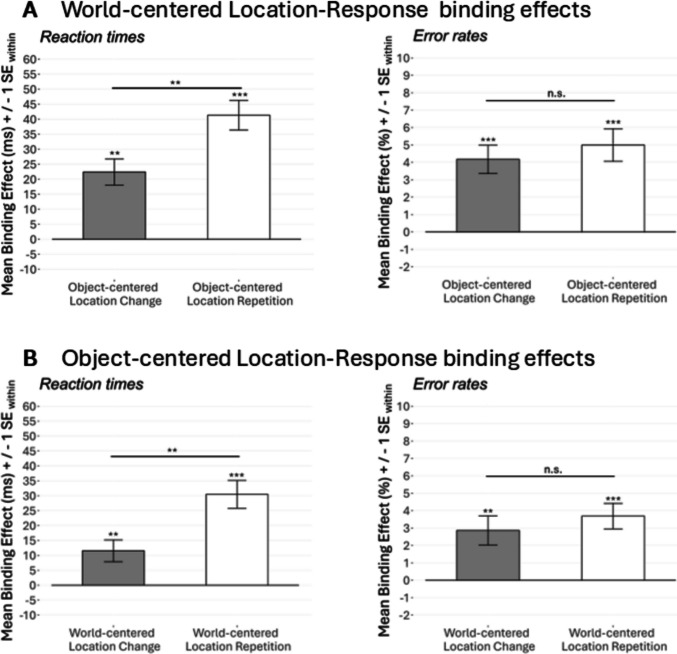
S-R binding effects in Experiment 2. *Note*. Panel **A** shows S-R binding effects (reaction times and error rates) for world-centered location in trials with object-centered location change and repetition. Panel **B** shows S-R binding effects for object-centered location in trials with world-centered location change and repetition. See Appendices 2B & 2 F for a depiction of all single data points that contribute to the binding effects. n.s. = *p* > 0.050, *p < 0.050, **p < 0.010, ***p < 0.001

The two-way interaction between world-centered location relation and object-centered location relation reached significance, *F*(1, 48) = 10.58, *p* = 0.002, ${{\upeta }_{G}}^{2}$ < 0.01, ${{\upeta }_{p}}^{2}$ = 0.18, as did the three-way interaction between response relation, world-centered location relation, and object-centered location relation: *F*(1, 48) = 10.27, *p* = 0.002, ${{\upeta }_{G}}^{2}$ < 0.01, ${{\upeta }_{p}}^{2}$ = 0.18. This indicates that, as in Experiment 1, the strength of the S-R binding effect_object-centered_ was influenced by whether the world-centered location was repeated or changed (and vice-versa, the pattern for the S-R binding effect_world-centered_ can be seen in Fig. 6A).

As in Experiment 1, we conducted exploratory post hoc *t*-tests using the calculated S-R binding effects, to take a closer look at the pattern of the S-R binding effect_object-centered_. Here, unlike in Experiment 1, we observed significant S-R binding effects for object-centered location in both trials with world-centered location change and world-centered location repetition. Particularly interesting here was the S-R binding effect for object-centered location when the world-centered location was *repeated* (because in this case, the absolute position of the stimulus between prime and probe did not change): A *t* test against zero revealed a significant S-R binding effect for object-centered location in trials in which the world-centered location was repeated: two-tailed,* t(48) = 6.54, p* < 0.001, *d*_z_ = 0.93, BF_01_ < 0.01 (*M* = 30 ms, *SD* = 33 ms, see Fig. 6B). This effect indicates that even when the absolute stimulus position did not change from prime to probe, an S-R binding effect for object-centered location occurred.

Finally, also in trials, in which the world-centered location changed, we found a significant binding effect for object-centered location (*M* = 12 ms, *SD* = 26 ms): against zero, two-tailed* t(48) = 3.16, p* = 0.003, *d*_z_ = 0.45, BF_01_ = 0.09. As indicated by the significant three-way interaction (see above), this S-R binding effect for object-centered location was significantly lower than in trials in which the world-centered location was repeated: two-tailed* t(47) = 3.20, p* = 0.002, *d*_z_ = 0.46, BF_01_ = 0.08. However, it indicates that S-R binding for object-centered location also occurred when the world-centered location changed.

#### Error rates

As for reaction times, a 2 (response relation: repetition vs. change) × 2 (world-centered location relation: repetition vs. change) × 2 (object-centered location relation: repetition vs. change) ANOVA on probe error rates was conducted (for the exploratory ANOVA output including animal identity relation, see Appendix 2H).

A significant two-way interaction was found between response relation and world-centered location relation, indicating S-R binding effects for response and world-centered location (overall binding effect_world-centered_): *F*(1, 48) = 49.28, *p* < 0.001, ${{\upeta }_{G}}^{2}$ = 0.06, ${{\upeta }_{p}}^{2}$ = 0.51.

Importantly, the two-way interaction between response relation and object-centered location relation (overall binding effect_object-centered_) reached significance as well, *F*(1, 48) = 38.60, *p* < 0.001, ${{\upeta }_{G}}^{2}$ = 0.03, ${{\upeta }_{p}}^{2}$ = 0.45, confirming the pattern found in the reaction times (i.e., overall S-R binding effect_object-centered_).

Furthermore, neither the two-way interaction between world-centered location relation and object-centered location relation reached significance, *F*(1, 48) = 1.40, *p* = 0.243, ${{\upeta }_{G}}^{2}$ < 0.01, ${{\upeta }_{p}}^{2}$ = 0.03, nor the three-way interaction between response relation, world-centered location relation, and object-centered location relation: *F*(1, 48) = 0.49, *p* = 0.486, ${{\upeta }_{G}}^{2}$ < 0.01, ${{\upeta }_{p}}^{2}$ = 0.01. This indicates that S-R binding effects occurred for object-centered location *independently* of the prime–probe relation of world-centered location. This supports the findings observed in the reaction times: S-R binding effects for object-centered location were found even when the absolute position of the target stimulus did not change at all.

### Discussion

The findings from Experiment 2 indicate that relative (i.e., object-centered) stimulus locations were bound to the response in both trials with a repetition in world-centered location (in which the absolute stimulus position did not change from prime to probe) as well as in trials with a change in world-centered location. Unlike Experiment 1, where all significant S-R binding effects could be explained by changes in the stimulus’ absolute position on the screen, Experiment 2 showed evidence for S-R binding effects solely attributable to relative (i.e., object-centered) location. This binding of relative positions appears to be adaptive, as the absolute positions were no longer sufficient to clearly define the information about the location of the target stimulus. Also, the fact that there were only four (and not 16) absolute positions at which the target stimulus could appear may have had an enhancing effect on the processing of relative positions in that more processing/attentional capacity was available.

Furthermore, as already observed in Experiment 1, absolute (i.e., world-centered) locations were bound to the response but influenced by the object-centered location relation: S-R binding effects were smaller when the object-centered location changed compared to trials in which the object-centered location was repeated (at least in reaction times). This supports the idea of a gradient in stimulus feature similarity that is reflected in S-R binding effects: The less similar prime and probe stimulus features are, the smaller S-R binding effects become (e.g., Schöpper et al., 2020; Singh et al., 2016).

## General discussion

In this study, the location of a target stimulus could be assessed in terms of two different frames of reference: Through its absolute position on the display (i.e., world-centered frame of reference) or through its relative position within an object (i.e., object-centered frame of reference). This was operationalized by using an object divided into four squares, which could appear at one of four display quadrants. The target stimulus was located at a) a relative position nested inside the object and b) an absolute position on the display. Stimulus–response (S-R) binding effects could be calculated for both types of stimulus locations independently.

In Experiment 1, the world-centered location was applied to the object’s center (‘object-locked’). Consequently, the target stimulus could appear at one of 16 possible absolute positions, one for each possible trial type (as a function of world-centered location and object-centered location). Therefore, only in trials with world-centered location change, we were able to attribute possible S-R binding effects for object-centered location specifically to the relative position within the object (because in all other trial types, location changes/repetitions corresponded to the repetition/change of absolute positions on the display).

With 16 absolute target stimulus positions, S-R binding effects could be found for the target stimulus’ world-centered location and, importantly, for object-centered location, but only in trials with world-centered location repetition. Therefore, the ‘purely’ relative location—that is, object-centered location in trials with world-centered location change—did not yield S-R binding effects. It rather seems likely that only the changes and repetitions of absolute positions caused S-R binding effects in Experiment 1: Since every possible target stimulus had a unique absolute position on the display, the cognitive system could define every target stimulus’ location conclusively, independent of world-centered (nested) or object-centered frames of reference. We assume that the relative stimulus positions simply did not provide any additional information value and that the processing thus had no benefit. So far, this is in line with common studies examining (absolute) stimulus locations in S-R binding (e.g., Frings & Moeller, 2010; Hommel, 1998).

As further exploratory analyses showed, S-R binding effects for world-centered location were modulated by object-centered location relation. In trials with world-centered location repetition and object-centered location change, the target stimulus re-appeared in the same quadrant, but in a slightly different absolute position: Interestingly, S-R binding effects got weaker in this case (compared with trials with object-centered location repetition), but did not disappear. Absolute stimulus positions, which deviated ‘slightly’ from each other, were still processed as (weaker) stimulus feature *repetitions*. This fits in with findings that perceptually similar stimuli still cause retrieval and thus S-R binding effects, which, however, gradually decrease with increasing deviation (Münster et al., 2024; Schöpper et al., 2020; Singh et al., 2016).

Critically, as mentioned above, we observed S-R binding effects for object-centered location in trials with world-centered location repetition. Here, the very same ‘slight’ deviance of absolute stimulus positions was apparently processed as a stimulus feature *change* (as otherwise there should be no S-R binding effects in this condition). Hence, it seems that it depends on the stimulus’ context (here: the frame of reference) whether a certain deviation is processed as a (gradually weaker) stimulus feature *repetition* or as a stimulus feature *change*. We conclude that assessing the stimulus’ location on a rather coarse (i.e., world-centered) or a more finely resolved (i.e., object-centered) frame of reference causes different processing of the very same absolute deviance (cf. Münster & Frings, in press, for context-dependent evaluation of stimulus feature relations).

In Experiment 2, we tested whether relative stimulus positions would be used in binding and retrieval processes when absolute stimulus positions were not unique (not ‘conclusive’) for every possible relative position/object-centered location. Therefore, the world-centered location was applied to the target stimulus (‘target stimulus-locked’), resulting in only four absolute positions where the target stimulus could appear. Therefore, the absolute position of the target stimulus was always repeated in trials with world-centered location repetition, independently of changes in object-centered location. Consequently, in trials with object-centered location change, the position of the target stimulus only changed in a ‘purely’ relative way within the object and not in terms of absolute coordinates. The four absolute target stimulus positions were therefore *not* unique for all possible target stimulus locations (i.e., a total of 16 as a function of world-centered locations and object-centered locations, as in Experiment 1).

We found S-R binding effects emerging for both types of locations in Experiment 2. Importantly, object-centered location yielded S-R binding effects in trials with world-centered location change (where the target stimulus’ absolute position *always* changed) and in trials with world-centered location repetition (where the target stimulus’ absolute position *never* changed). This indicates S-R bindings of ‘purely’ relative positions.

Together with the results from Experiment 1, this suggests that when there are different ways available to conclusively determine the location of a stimulus, the absolute position of the stimulus is prioritized over its relative position (which may be redundant as it does not contain any additional information that is not provided by absolute positions). Only when absolute positions are no longer conclusive—that is, when absolute positions no longer contain all the information about the (relative) location of a stimulus—nested relative positions are processed and used for binding and retrieval.

Finally, in an exploratory analysis, Experiment 2 showed the same modulation of binding effects for world-centered location by object-centered location relation as Experiment 1 (in reaction times). This supports the idea that deviations of absolute positions are processed differently in different contexts (here: frames of reference): Either as (gradually weaker) repetitions or as changes. This makes sense from an ecological point of view: The location of a stimulus within the world (i.e., the four quadrants in the present study), for example, the position of a clock within the room, should be less sensitive to relatively small deviations. Nested locations, by contrast, take place in an objectively much smaller space, where these small deviations make a much greater difference: If you want to read the clock, it is important to be able to define exactly where the clock hands are on the clock face.

Given the stimulus configuration used in our paradigm, it is very likely that participants performed saccades prior to color classification and response execution. However, no eye-movement data were recorded in the present study. Though it is known from the literature that responding with eye movements is bound and retrieved like responses carried out with other effectors (e.g., fingers; Schöpper et al., 2022), we can only speculate about the role that saccades play in the creation of stimulus location-response bindings. Upcoming studies should thus investigate the potential interaction of saccadic landing positions and key-press responses in location-response binding tasks.

## Conclusion

The present study examined how the availability of absolutely unique coordinates modulated the use of the nested, relative stimulus location for binding and retrieval processes. Our results suggest that location may not be a singular stimulus feature but has multiple facets comparable to other stimulus features like color (e.g., luminance, hue; Laub & Frings, 2021), or words (e.g., meaning, word type; Singh et al., 2018) that can be *separately* bound to a response.

We conclude that relative stimulus locations are *only* used for binding and retrieval processes when the ‘where’ of the stimulus cannot be clearly defined only by its absolute position (i.e., when one absolute position contains more than one relative position). Whether the relevance of a particular frame of reference (e.g., through additional tasks) can modulate its use in binding and retrieval processes was not the scope of this study and should be investigated in the future. At this point, it is evident that the various available location information can be used adaptively for action control processes. It seems that the set of stimulus features used in a task influences how their relation (here: the deviation in the stimulus’ location) is evaluated; as rather a feature repetition or feature change.

## Supplementary Information

Below is the link to the electronic supplementary material.

Supplementary file1 (DOCX 475 KB)

## Data Availability

The data for all experiments are available at OSF (https://osf.io/qpytx/?view_only=be9804b3106a482196213c6746a6ba92). Materials are not available, as they are described in detail in the text and can therefore be easily reproduced.

## Footnotes

1 For the sake of distinctiveness, we speak of location as the coded stimulus feature (cf. Frings et al., 2024)—each frame of reference (here: object-centered/world-centered) thus results in another location feature. Position, on the other hand, refers to the physical, objective component of a stimulus in space (here: absolute coordinates on the display/relative relation to context stimuli).

2 Regarding our analyses, S-R binding effects are indicated by the interactions between response relation and stimulus feature relation and the corresponding *t* tests against zero—since our study addresses the question of the occurrence of S-R binding effects per se, any further modulations and interactions are therefore to be understood exploratively.

3 One of the excluded participants could not be included in the analyses because of missing conditions due to too few correctly answered trials. For the other excluded participant, trials from all conditions were available, but the error rate was above 50% (indicating a nonunderstanding of the task instructions). Therefore, participants were not excluded as outliers, but they were unfit for analyses.

4 By using two different animals, we were able to *exploratively* add animal identity relation as a factor to a 2 (response relation: repetition vs. change) × 2 (world-centered location relation: repetition vs. change) × 2 (object-centered location relation: repetition vs. change) × 2 (animal identity relation: repetition vs. change) ANOVA, in order to detect possible S-R binding effects for the animal identity. In fact, we found this S-R binding effect (i.e., the interaction between response relation and animal identity relation) in reaction times, *F*(1, 50) = 31.37, *p* <.001, ${{\upeta }_{G}}^{2}$ < 0.01, ${{\upeta }_{p}}^{2}$ = 0.39, as well as in error rates, *F*(1, 50) = 10.48, *p* =.002, ${{\upeta }_{G}}^{2}$ = 0.01, ${{\upeta }_{p}}^{2}$ = 0.17. However, the factor distractor identity relation interacted with none of the other factors (i.e., world-centered location relation, and object-centered location relation) in both the reaction time and the error rates. Although it is interesting that the classic distractor–response binding effect for distractor animal identity occurred independently of location repetitions or changes, the factor animal identity relation was not considered in our hypotheses regarding the S-R binding effects for stimulus locations. Therefore, the factor animal identity relation was not included in the ANOVA that was computed in the main Results section. For a comparison of ANOVAs with and without the factor animal identity relation, see Appendices 1 C & 1D (reaction times) and Appendices 1G & 1H (error rates).

5 Importantly, the animals were not introduced as task-relevant stimuli. The text in the instruction, that followed the explanation of the colored frame, was: ‘In dem Rahmen wird immer ein Tier zu sehen sein: Entweder ist das Emil, der Elefant, oder Niko, das Nilpferd.’/‘There will always be an animal in the frame: either Emil, the elephant, or Niko, the hippopotamus.’

6 It was explained to the participants in the instruction that each trial started with a fixation cross and that the star seen during the trials only served to fixate the center of the screen.

7 Note that calculating a S-R binding effect primarily simplifies the analysis processes because simple *t* tests can be calculated, instead of an ANOVA + post hoc tests or contrasts. Furthermore, modulations of or differences between binding effects can be more easily and more comprehensibly analyzed and graphically represented in this way. All single data points used in the formula can be found in Appendices 1 A (reaction times) and 1E (error rates) for Experiment 1 and 2 A (reaction times) and 2E (error rates) for Experiment 2.

8 One participant could not be included in the analyses because of missing conditions due to too few correctly answered trials. When performing the analyses including the three outliers, the effects only differed in terms of their strength, except for one substantial difference: In reaction times, the difference in S-R binding effects_object-centered_ between the two stages of world-centered location changed from BF_01_ = 0.08 to BF_01_ = 0.33 (with *p* values changing from *p* =.002 to *p* =.013).

9 As in Experiment 1, this allowed us to exploratively look at S-R binding effects for animal identity. We found this S-R binding effect (i.e., the interaction with response relation) in reaction times, *F*(1, 48) = 18.12, *p* <.001, ${{\upeta }_{G}}^{2}$ < 0.01, ${{\upeta }_{p}}^{2}$ = 0.27, as well as in error rates, *F*(1, 48) = 5.81, *p* =.020, ${{\upeta }_{G}}^{2}$ = 0.01, ${{\upeta }_{p}}^{2}$ = 0.11. Again, the factor distractor identity interacted with none of the other factors (i.e., world-centered location relation, and object-centered location relation) in both the reaction time and the error rates ANOVA. For a comparison of ANOVAs with and without the factor animal identity relation, see Appendices 2 C & 2D (reaction times) and Appendices 2G & 2H (error rates).

## Code availability

Analysis scripts and codes are not available online (but can be provided on request), as all analysis steps are described in detail in the text and can therefore be easily reproduced.

## Declarations

### Author notes

The Deutsche Forschungsgemeinschaft supported the research reported in this article (FR2133/18–1).

### Open practices statement

The data for all experiments are available at OSF (https://osf.io/qpytx/?view_only=be9804b3106a482196213c6746a6ba92). None of the experiments was preregistered.
