# Supplementary material for: It’s about location, location, location: Absolute and relative stimulus positions in action control
Source: Atten Percept Psychophys. 2025 Apr 24;87(5):1678–93. doi: 10.3758/s13414-025-03062-1 (PMC12204904; doi:10.3758/s13414-025-03062-1)
Supplement: Supplementary file 1 — Supplementary file1 (DOCX 475 KB) [file 13414_2025_3062_MOESM1_ESM.docx]

**Appendix 1A**

*Descriptive statistics (reaction times) for Experiment 1.*

| Response Relation | Object-centered Location Relation | World-centered Location Relation | Animal Identity Relation | Mean Reaction Times (ms) |
| --- | --- | --- | --- | --- |
| CHANGE | CHANGE | CHANGE | CHANGE | 573.84 |
| CHANGE | CHANGE | CHANGE | REPETITION | 577.25 |
| CHANGE | CHANGE | REPETITION | CHANGE | 569.41 |
| CHANGE | CHANGE | REPETITION | REPETITION | 572.81 |
| CHANGE | REPETITION | CHANGE | CHANGE | 572.81 |
| CHANGE | REPETITION | CHANGE | REPETITION | 578.70 |
| CHANGE | REPETITION | REPETITION | CHANGE | 571.04 |
| CHANGE | REPETITION | REPETITION | REPETITION | 573.42 |
| REPETITION | CHANGE | CHANGE | CHANGE | 566.19 |
| REPETITION | CHANGE | CHANGE | REPETITION | 553.19 |
| REPETITION | CHANGE | REPETITION | CHANGE | 541.64 |
| REPETITION | CHANGE | REPETITION | REPETITION | 531.52 |
| REPETITION | REPETITION | CHANGE | CHANGE | 563.26 |
| REPETITION | REPETITION | CHANGE | REPETITION | 554.60 |
| REPETITION | REPETITION | REPETITION | CHANGE | 518.07 |
| REPETITION | REPETITION | REPETITION | REPETITION | 501.19 |

**Appendix 1B**

*Line plots showing the data points (reaction times) for world-centered location relation and response relation for the two stages of object-centered location relation (A) and for object-centered location relation and response relation for the two stages of world-centered location relation (B) in Experiment 1.*

**
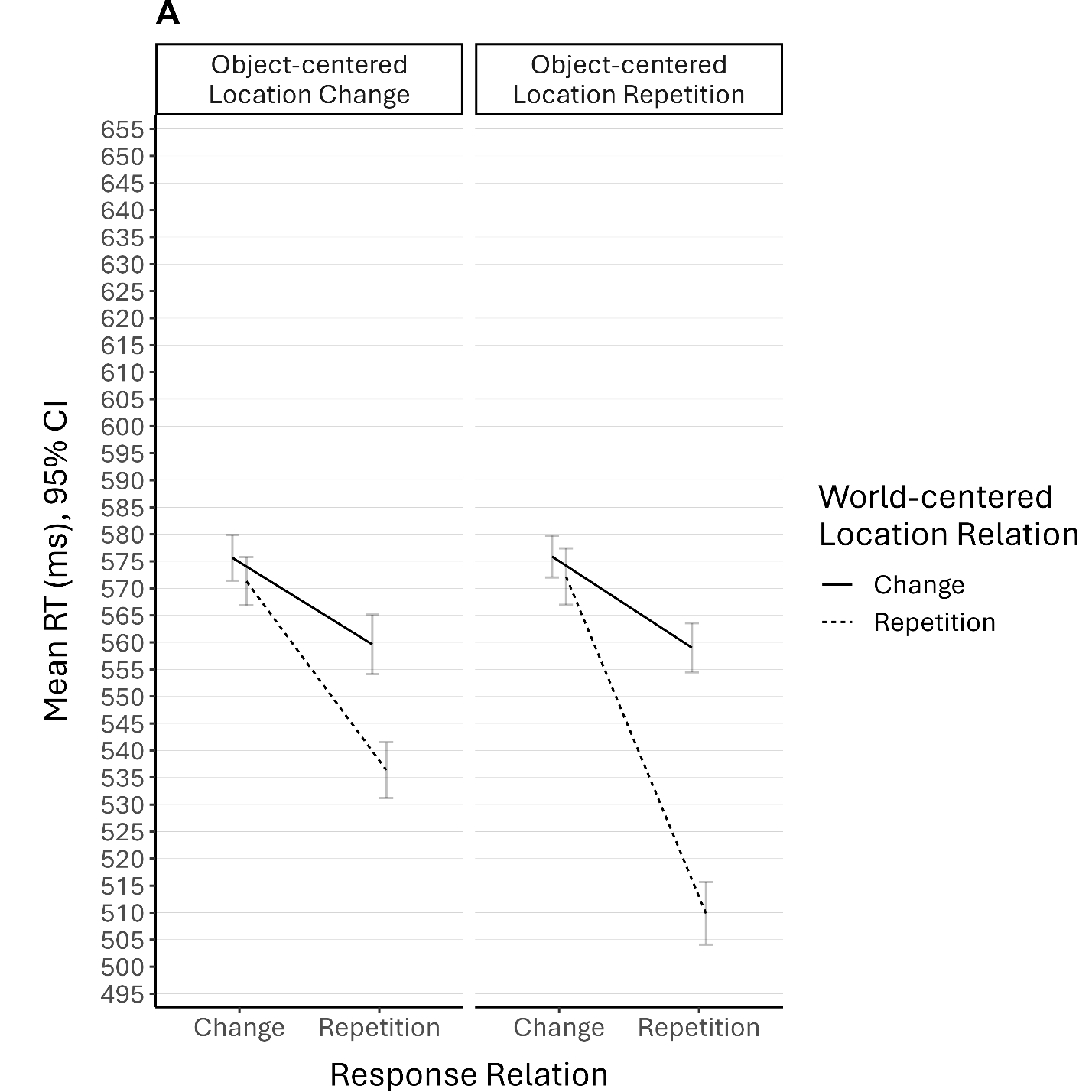
**

**
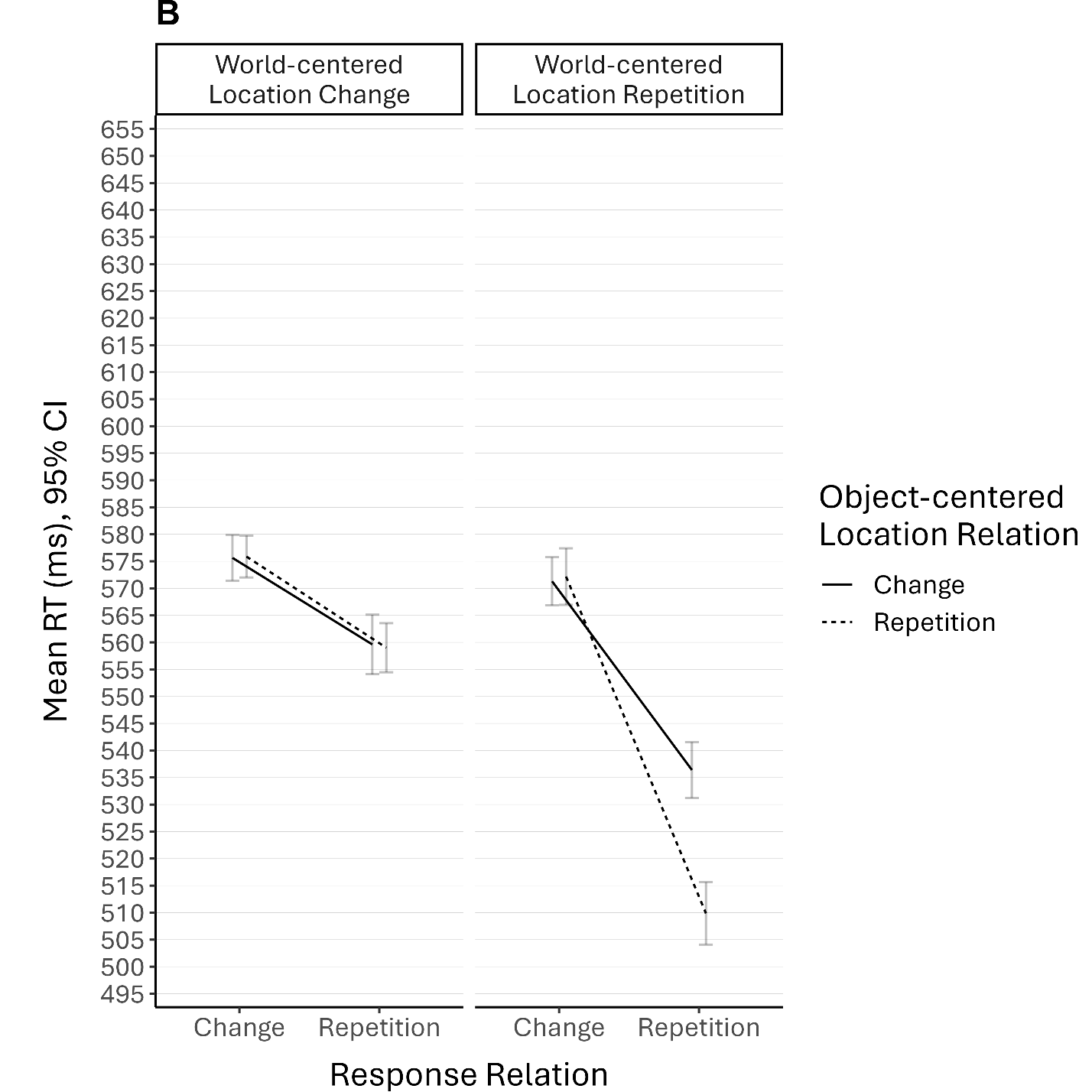
**

**Appendix 1C**

*ANOVA results (reaction times) for Experiment 1 without the factor ‘animal identity relation’.*

| Predictor | *df_Num_* | *df_Den_* | *F* | *p* | η^2^_p_ |
| --- | --- | --- | --- | --- | --- |
| (Intercept) | 1 | 50 | 2929.65 | .000 | .98 |
| Response Relation | 1 | 50 | 157.57 | .000 | .76 |
| Object-centered Location Relation | 1 | 50 | 21.00 | .000 | .30 |
| World-centered Location Relation | 1 | 50 | 93.88 | .000 | .65 |
| Response Relation x Object-centered Location Relation | 1 | 50 | 33.45 | .000 | .40 |
| Response Relation x World-centered Location Relation | 1 | 50 | 105.78 | .000 | .68 |
| Object-centered Location Relation x World-centered Location Relation | 1 | 50 | 20.32 | .000 | .29 |
| Response Relation x Object-centered Location Relation x World-centered Location Relation | 1 | 50 | 24.81 | .000 | .33 |

*Note.* *df_Num_* indicates degrees of freedom numerator. *df_Den_* indicates degrees of freedom denominator. η^2^_p_ indicates partial eta-squared.

**Appendix 1D**

*ANOVA results (reaction times) for Experiment 1 with the factor ‘animal identity relation’ included.*

| Predictor | *df_Num_* | *df_Den_* | *F* | *p* | η^2^_p_ |
| --- | --- | --- | --- | --- | --- |
| (Intercept) | 1 | 50 | 2935.71 | .000 | .98 |
| Response Relation | 1 | 50 | 156.26 | .000 | .76 |
| Object-centered Location Relation | 1 | 50 | 22.62 | .000 | .31 |
| World-centered Location Relation | 1 | 50 | 93.55 | .000 | .65 |
| Animal Identity Relation | 1 | 50 | 18.19 | .000 | .27 |
| Response Relation x Object-centered Location Relation | 1 | 50 | 40.65 | .000 | .45 |
| Response Relation x World-centered Location Relation | 1 | 50 | 107.69 | .000 | .68 |
| Object-centered Location Relation x World-centered Location Relation | 1 | 50 | 20.26 | .000 | .29 |
| Response Relation x Animal Identity Relation | 1 | 50 | 31.37 | .000 | .39 |
| Object-centered Location Relation x Animal Identity Relation | 1 | 50 | 0.01 | .927 | .00 |
| World-centered Location Relation x Animal Identity Relation | 1 | 50 | 0.68 | .412 | .01 |
| Response Relation x Object-centered Location Relation x World-centered Location Relation | 1 | 50 | 23.77 | .000 | .32 |
| Response Relation x Object-centered Location Relation x Animal Identity Relation | 1 | 50 | 0.09 | .769 | .00 |
| Response Relation x World-centered Location Relation x Animal Identity Relation | 1 | 50 | 0.03 | .869 | .00 |
| Object-centered Location Relation x World-centered Location Relation x Animal Identity Relation | 1 | 50 | 1.94 | .170 | .04 |
| Response Relation x Object-centered Location Relation x World-centered Location Relation x Animal Identity Relation | 1 | 50 | 0.69 | .411 | .01 |

*Note.* *df_Num_* indicates degrees of freedom numerator. *df_Den_* indicates degrees of freedom denominator. η^2^_p_ indicates partial eta-squared.

**Appendix 1E**

*Descriptive statistics (error rates) for Experiment 1.*

| Response Relation | Object-centered Location Relation | World-centered Location Relation | Animal Identity Relation | Mean Error Rates (%) |
| --- | --- | --- | --- | --- |
| CHANGE | CHANGE | CHANGE | CHANGE | 6.12 |
| CHANGE | CHANGE | CHANGE | REPETITION | 8.08 |
| CHANGE | CHANGE | REPETITION | CHANGE | 6.88 |
| CHANGE | CHANGE | REPETITION | REPETITION | 7.88 |
| CHANGE | REPETITION | CHANGE | CHANGE | 6.40 |
| CHANGE | REPETITION | CHANGE | REPETITION | 6.36 |
| CHANGE | REPETITION | REPETITION | CHANGE | 7.63 |
| CHANGE | REPETITION | REPETITION | REPETITION | 10.47 |
| REPETITION | CHANGE | CHANGE | CHANGE | 7.47 |
| REPETITION | CHANGE | CHANGE | REPETITION | 7.20 |
| REPETITION | CHANGE | REPETITION | CHANGE | 6.26 |
| REPETITION | CHANGE | REPETITION | REPETITION | 6.06 |
| REPETITION | REPETITION | CHANGE | CHANGE | 7.61 |
| REPETITION | REPETITION | CHANGE | REPETITION | 6.86 |
| REPETITION | REPETITION | REPETITION | CHANGE | 3.65 |
| REPETITION | REPETITION | REPETITION | REPETITION | 4.22 |

**Appendix 1F**

*Line plots showing the data points (error rates) for world-centered location relation and response relation for the two stages of object-centered location relation (A) and for object-centered location relation and response relation for the two stages of world-centered location relation (B) in Experiment 2.*

**
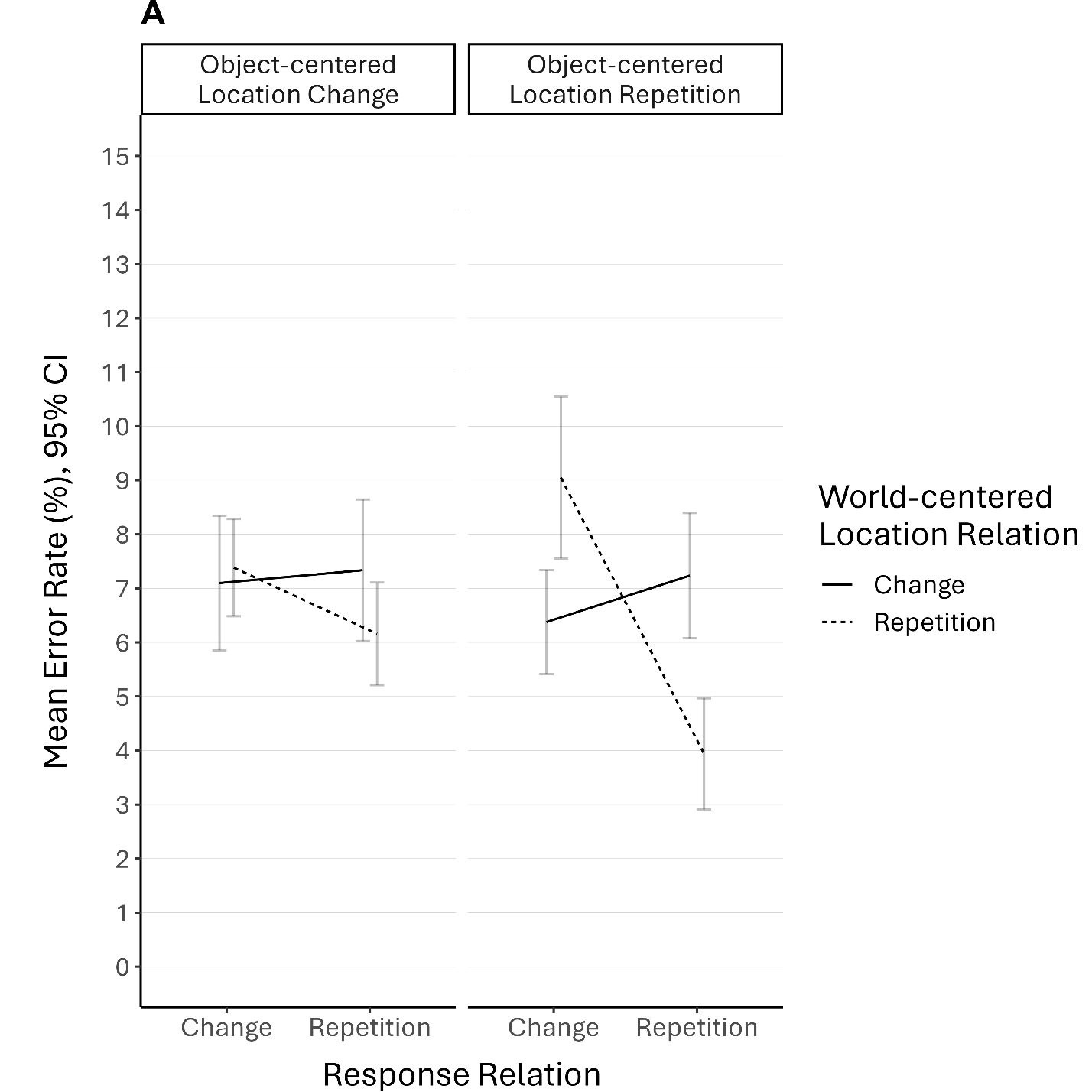
**

**
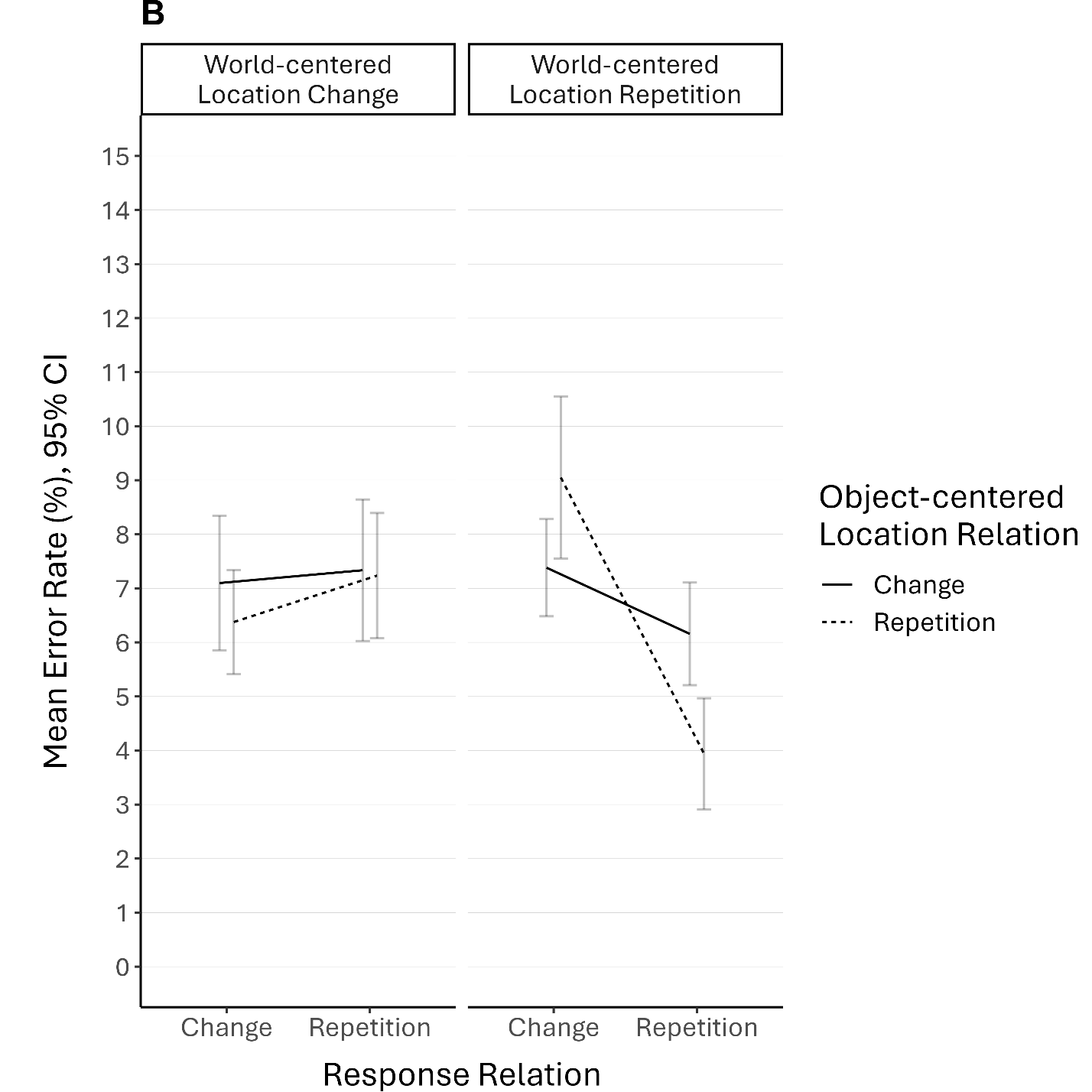
**

**Appendix 1G**

*ANOVA results (error rates) for Experiment 1 without the factor ‘animal identity relation’.*

| Predictor | *df_Num_* | *df_Den_* | *F* | *p* | η^2^_p_ |
| --- | --- | --- | --- | --- | --- |
| (Intercept) | 1 | 50 | 90.04 | .000 | .64 |
| Response Relation | 1 | 50 | 3.01 | .089 | .06 |
| Object-centered Location Relation | 1 | 50 | 1.29 | .261 | .03 |
| World-centered Location Relation | 1 | 50 | 1.17 | .285 | .02 |
| Response Relation x Object-centered Location Relation | 1 | 50 | 17.56 | .000 | .26 |
| Response Relation x World-centered Location Relation | 1 | 50 | 19.52 | .000 | .28 |
| Object-centered Location Relation x World-centered Location Relation | 1 | 50 | 0.82 | .371 | .02 |
| Response Relation x Object-centered Location Relation x World-centered Location Relation | 1 | 50 | 14.02 | .000 | .22 |

*Note.* *df_Num_* indicates degrees of freedom numerator. *df_Den_* indicates degrees of freedom denominator. η^2^_p_ indicates partial eta-squared.

**Appendix 1H**

*ANOVA results (error rates) for Experiment 1 with the factor ‘animal identity relation’ included.*

| Predictor | *df_Num_* | *df_Den_* | *F* | *p* | η^2^_p_ |
| --- | --- | --- | --- | --- | --- |
| (Intercept) | 1 | 50 | 90.04 | .000 | .64 |
| Response Relation | 1 | 50 | 3.01 | .089 | .06 |
| Object-centered Location Relation | 1 | 50 | 1.29 | .261 | .03 |
| World-centered Location Relation | 1 | 50 | 1.17 | .285 | .02 |
| Animal Identity Relation | 1 | 50 | 4.32 | .043 | .08 |
| Response Relation x Object-centered Location Relation | 1 | 50 | 17.56 | .000 | .26 |
| Response Relation x World-centered Location Relation | 1 | 50 | 19.52 | .000 | .28 |
| Object-centered Location Relation x World-centered Location Relation | 1 | 50 | 0.82 | .371 | .02 |
| Response Relation x Animal Identity Relation | 1 | 50 | 10.47 | .002 | .17 |
| Object-centered Location Relation x Animal Identity Relation | 1 | 50 | 0.04 | .833 | .00 |
| World-centered Location Relation x Animal Identity Relation | 1 | 50 | 0.23 | .630 | .00 |
| Response Relation x Object-centered Location Relation x World-centered Location Relation | 1 | 50 | 14.02 | .000 | .22 |
| Response Relation x Object-centered Location Relation x Animal Identity Relation | 1 | 50 | 1.01 | .319 | .02 |
| Response Relation x World-centered Location Relation x Animal Identity Relation | 1 | 50 | 0.01 | .912 | .00 |
| Object-centered Location Relation x World-centered Location Relation x Animal Identity Relation | 1 | 50 | 1.06 | .309 | .02 |
| Response Relation x Object-centered Location Relation x World-centered Location Relation x Animal Identity Relation | 1 | 50 | 0.87 | .354 | .02 |

*Note.* *df_Num_* indicates degrees of freedom numerator. *df_Den_* indicates degrees of freedom denominator. η^2^_p_ indicates partial eta-squared.

**Appendix 2A**

*Descriptive statistics (reaction times) for Experiment 2.*

| Response Relation | Object-centered Location Relation | World-centered Location Relation | Animal Identity Relation | Mean Reaction Times (ms) |
| --- | --- | --- | --- | --- |
| CHANGE | CHANGE | CHANGE | CHANGE | 597.96 |
| CHANGE | CHANGE | CHANGE | REPETITION | 608.07 |
| CHANGE | CHANGE | REPETITION | CHANGE | 602.42 |
| CHANGE | CHANGE | REPETITION | REPETITION | 599.96 |
| CHANGE | REPETITION | CHANGE | CHANGE | 599.48 |
| CHANGE | REPETITION | CHANGE | REPETITION | 606.59 |
| CHANGE | REPETITION | REPETITION | CHANGE | 600.20 |
| CHANGE | REPETITION | REPETITION | REPETITION | 603.71 |
| REPETITION | CHANGE | CHANGE | CHANGE | 596.85 |
| REPETITION | CHANGE | CHANGE | REPETITION | 588.38 |
| REPETITION | CHANGE | REPETITION | CHANGE | 572.74 |
| REPETITION | CHANGE | REPETITION | REPETITION | 563.72 |
| REPETITION | REPETITION | CHANGE | CHANGE | 585.21 |
| REPETITION | REPETITION | CHANGE | REPETITION | 576.77 |
| REPETITION | REPETITION | REPETITION | CHANGE | 543.40 |
| REPETITION | REPETITION | REPETITION | REPETITION | 534.06 |

**Appendix 2B**

*Line plots showing the data points (reaction times) for world-centered location relation and response relation for the two stages of object-centered location relation (A) and for object-centered location relation and response relation for the two stages of world-centered location relation (B) in Experiment 2.*

**
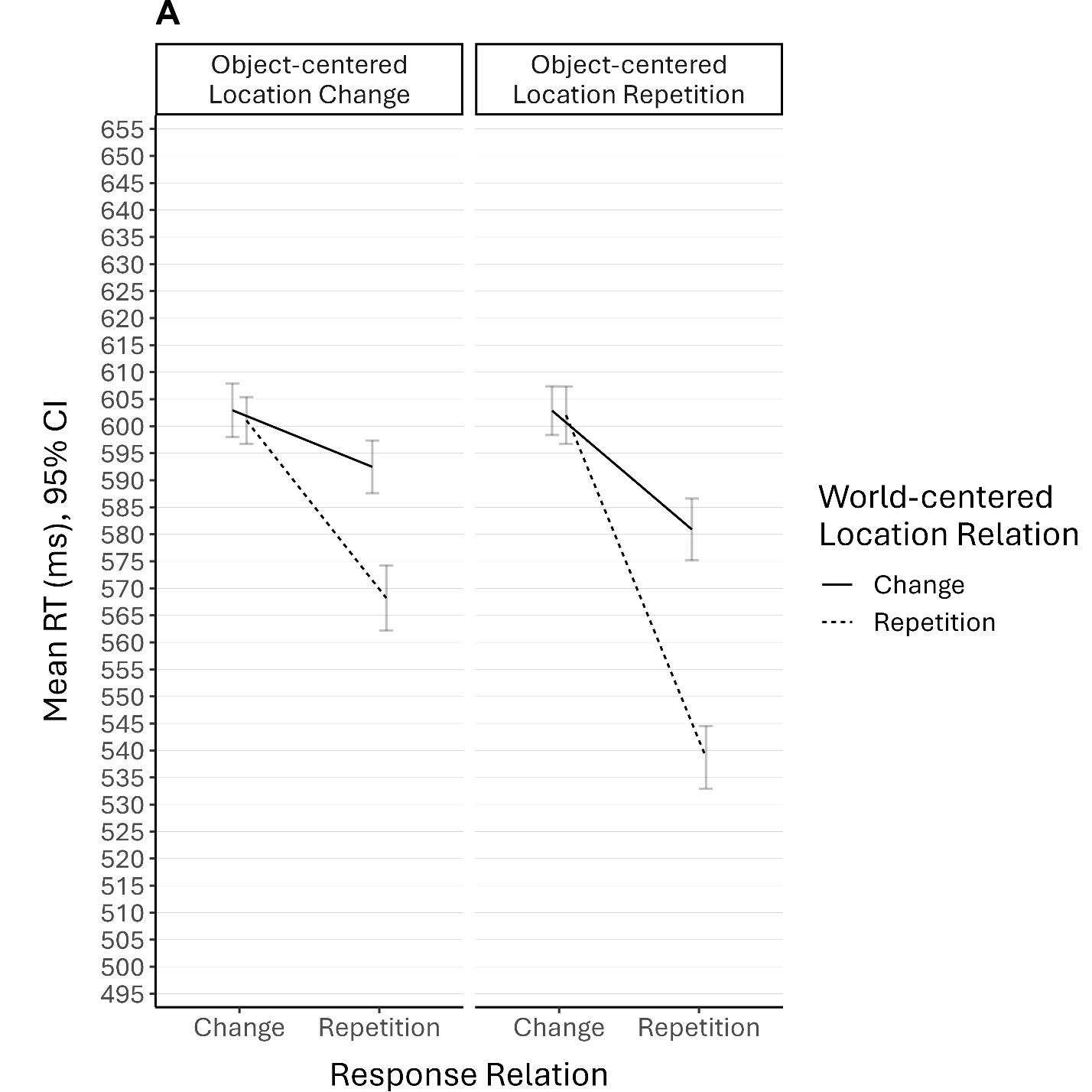
**

*
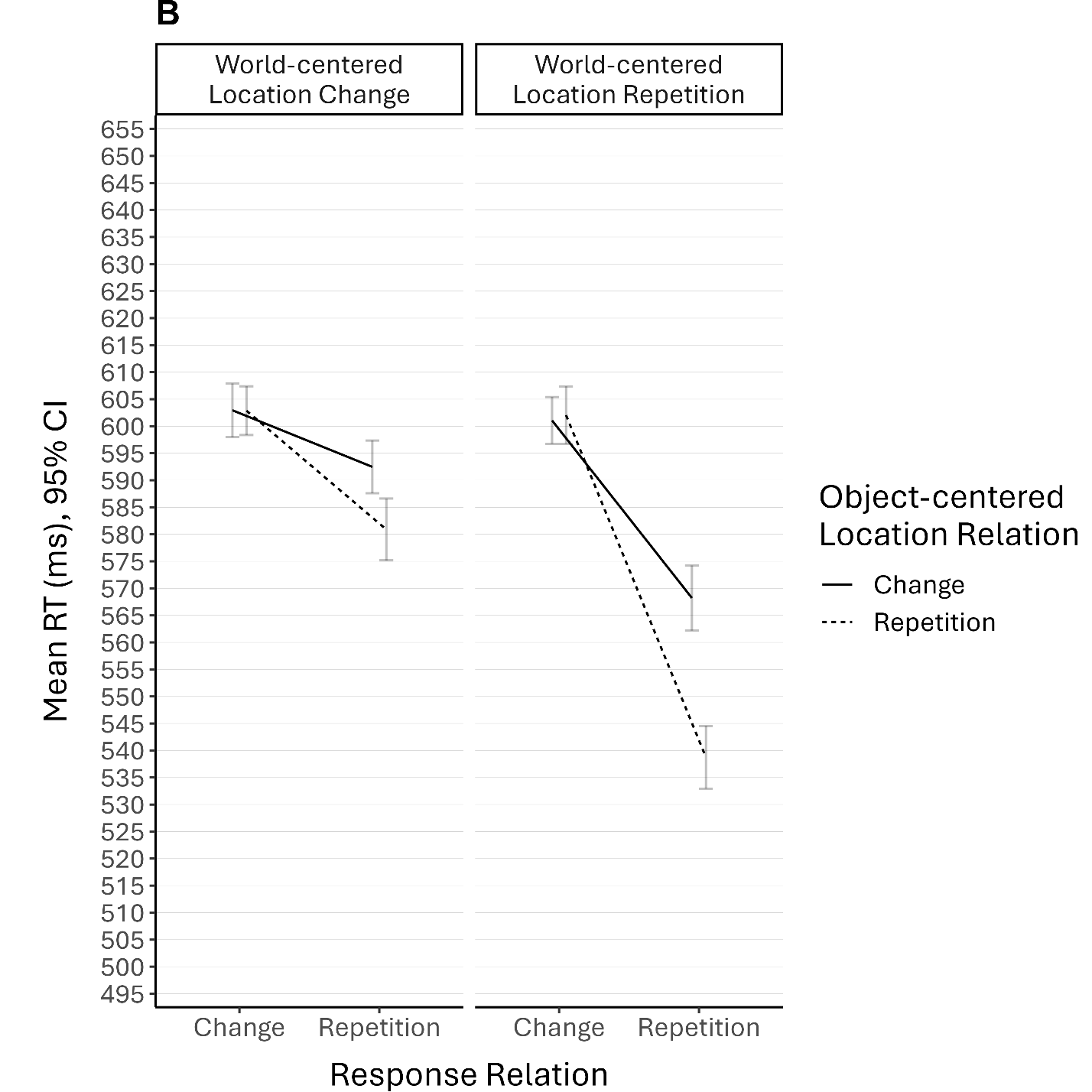
*

**Appendix 2C**

*ANOVA results (reaction times) for Experiment 2 without the factor ‘animal identity relation’.*

| Predictor | *df_Num_* | *df_Den_* | *F* | *p* | η^2^_p_ |
| --- | --- | --- | --- | --- | --- |
| (Intercept) | 1 | 48 | 1709.75 | .000 | .97 |
| Response Relation | 1 | 48 | 123.45 | .000 | .72 |
| Object-centered Location Relation | 1 | 48 | 32.28 | .000 | .40 |
| World-centered Location Relation | 1 | 48 | 104.09 | .000 | .68 |
| Response Relation x Object-centered Location Relation | 1 | 48 | 50.28 | .000 | .51 |
| Response Relation x World-centered Location Relation | 1 | 48 | 78.45 | .000 | .62 |
| Object-centered Location Relation x World-centered Location Relation | 1 | 48 | 10.58 | .002 | .18 |
| Response Relation x Object-centered Location Relation x World-centered Location Relation | 1 | 48 | 10.27 | .002 | .18 |

*Note.* *df_Num_* indicates degrees of freedom numerator. *df_Den_* indicates degrees of freedom denominator. η^2^_p_ indicates partial eta-squared.

**Appendix 2D**

*ANOVA results (reaction times) for Experiment 2 with the factor ‘animal identity relation’ included.*

| Predictor | *df_Num_* | *df_Den_* | *F* | *p* | η^2^_p_ |
| --- | --- | --- | --- | --- | --- |
| (Intercept) | 1 | 48 | 1708.16 | .000 | .97 |
| Response Relation | 1 | 48 | 122.18 | .000 | .72 |
| Object-centered Location Relation | 1 | 48 | 30.99 | .000 | .39 |
| World-centered Location Relation | 1 | 48 | 106.14 | .000 | .69 |
| Animal Identity Relation | 1 | 48 | 2.98 | .091 | .06 |
| Response Relation x Object-centered Location Relation | 1 | 48 | 50.86 | .000 | .51 |
| Response Relation x World-centered Location Relation | 1 | 48 | 78.18 | .000 | .62 |
| Object-centered Location Relation x World-centered Location Relation | 1 | 48 | 10.83 | .002 | .18 |
| Response Relation x Animal Identity Relation | 1 | 48 | 18.12 | .000 | .27 |
| Object-centered Location Relation x Animal Identity Relation | 1 | 48 | 0.05 | .818 | .00 |
| World-centered Location Relation x Animal Identity Relation | 1 | 48 | 2.60 | .113 | .05 |
| Response Relation x Object-centered Location Relation x World-centered Location Relation | 1 | 48 | 10.26 | .002 | .18 |
| Response Relation x Object-centered Location Relation x Animal Identity Relation | 1 | 48 | 0.12 | .733 | .00 |
| Response Relation x World-centered Location Relation x Animal Identity Relation | 1 | 48 | 2.37 | .130 | .05 |
| Object-centered Location Relation x World-centered Location Relation x Animal Identity Relation | 1 | 48 | 0.40 | .528 | .01 |
| Response Relation x Object-centered Location Relation x World-centered Location Relation x Animal Identity Relation | 1 | 48 | 0.83 | .368 | .02 |

*Note.* *df_Num_* indicates degrees of freedom numerator. *df_Den_* indicates degrees of freedom denominator. η^2^_p_ indicates partial eta-squared.

**Appendix 2E**

*Descriptive statistics (error rates) for Experiment 2.*

| Response Relation | Object-centered Location Relation | World-centered Location Relation | Animal Identity Relation | Mean Error Rates (%) |
| --- | --- | --- | --- | --- |
| CHANGE | CHANGE | CHANGE | CHANGE | 2.79 |
| CHANGE | CHANGE | CHANGE | REPETITION | 4.63 |
| CHANGE | CHANGE | REPETITION | CHANGE | 5.16 |
| CHANGE | CHANGE | REPETITION | REPETITION | 5.70 |
| CHANGE | REPETITION | CHANGE | CHANGE | 4.07 |
| CHANGE | REPETITION | CHANGE | REPETITION | 5.33 |
| CHANGE | REPETITION | REPETITION | CHANGE | 7.13 |
| CHANGE | REPETITION | REPETITION | REPETITION | 7.82 |
| REPETITION | CHANGE | CHANGE | CHANGE | 5.61 |
| REPETITION | CHANGE | CHANGE | REPETITION | 5.92 |
| REPETITION | CHANGE | REPETITION | CHANGE | 3.67 |
| REPETITION | CHANGE | REPETITION | REPETITION | 2.96 |
| REPETITION | REPETITION | CHANGE | CHANGE | 4.03 |
| REPETITION | REPETITION | CHANGE | REPETITION | 3.75 |
| REPETITION | REPETITION | REPETITION | CHANGE | 2.10 |
| REPETITION | REPETITION | REPETITION | REPETITION | 1.24 |

**Appendix 2F**

*Line plots showing the data points (error rates) for world-centered location relation and response relation for the two stages of object-centered location relation (A) and for object-centered location relation and response relation for the two stages of world-centered location relation (B) in Experiment 2.*


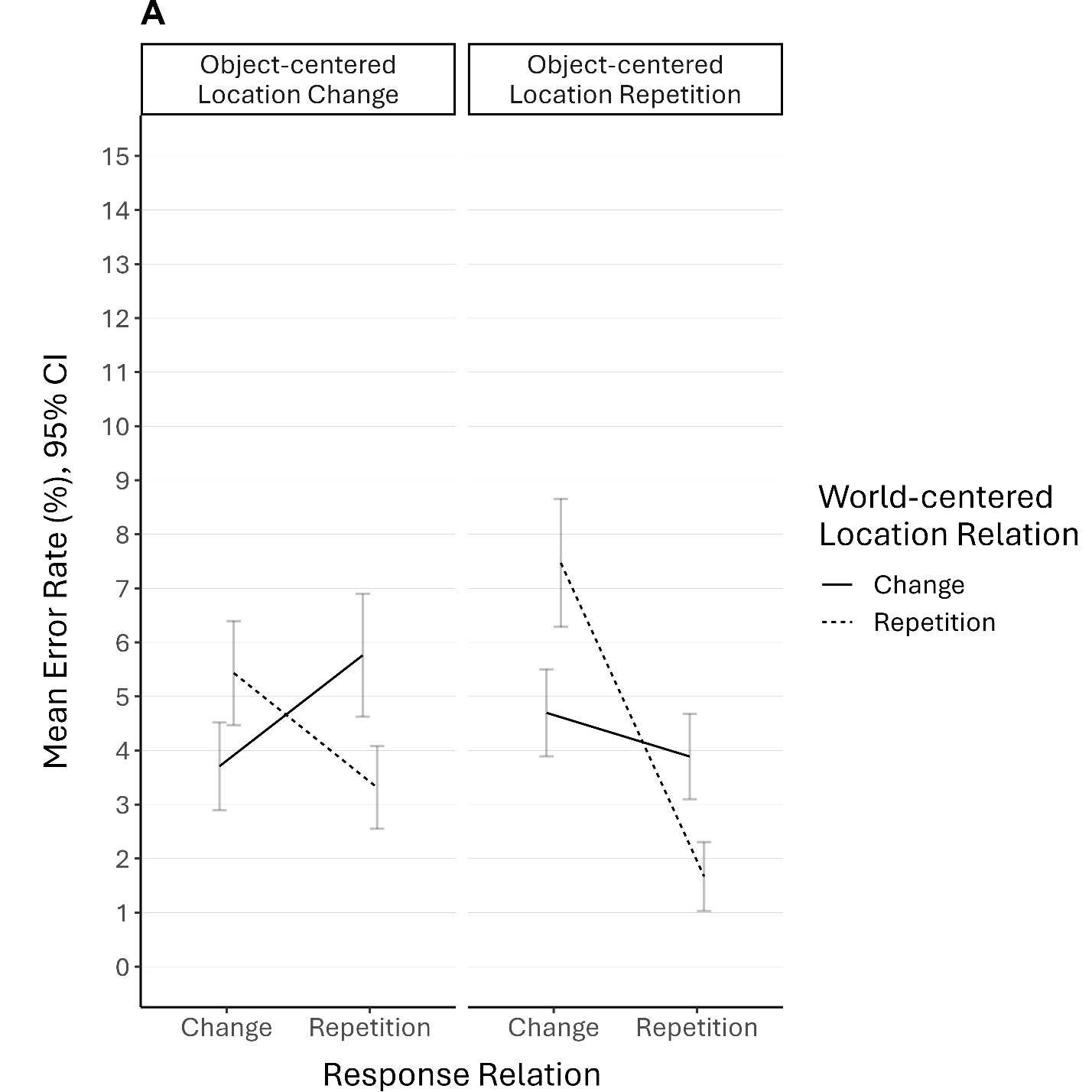


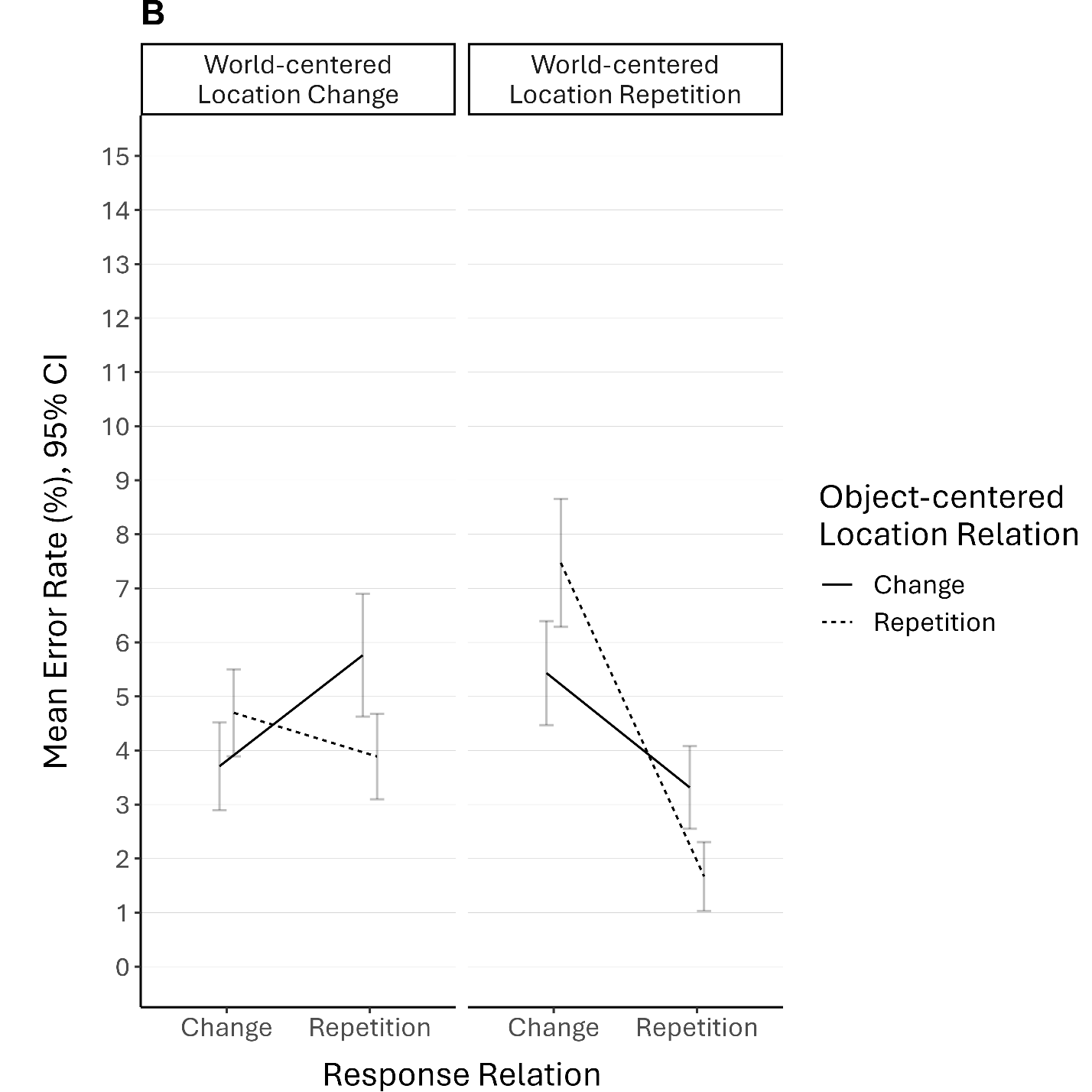


**Appendix 2G**

*ANOVA results (error rates) for Experiment 2 without the factor ‘animal identity relation’.*

| Predictor | *df_Num_* | *df_Den_* | *F* | *p* | η^2^_p_ |
| --- | --- | --- | --- | --- | --- |
| (Intercept) | 1 | 48 | 105.06 | .000 | .69 |
| Response Relation | 1 | 48 | 8.70 | .005 | .15 |
| Object-centered Location Relation | 1 | 48 | 0.17 | .686 | .00 |
| World-centered Location Relation | 1 | 48 | 0.02 | .903 | .00 |
| Response Relation x Object-centered Location Relation | 1 | 48 | 38.60 | .000 | .45 |
| Response Relation x World-centered Location Relation | 1 | 48 | 49.28 | .000 | .51 |
| Object-centered Location Relation x World-centered Location Relation | 1 | 48 | 1.40 | .243 | .03 |
| Response Relation x Object-centered Location Relation x World-centered Location Relation | 1 | 48 | 0.49 | .486 | .01 |

*Note.* *df_Num_* indicates degrees of freedom numerator. *df_Den_* indicates degrees of freedom denominator. η^2^_p_ indicates partial eta-squared.

**Appendix 2H**

*ANOVA results (error rates) for Experiment 2 with the factor ‘animal identity relation’ included.*

| Predictor | *df_Num_* | *df_Den_* | *F* | *p* | η^2^_p_ |
| --- | --- | --- | --- | --- | --- |
| (Intercept) | 1 | 48 | 105.06 | .000 | .69 |
| Response Relation | 1 | 48 | 8.70 | .005 | .15 |
| Object-centered Location Relation | 1 | 48 | 0.17 | .686 | .00 |
| World-centered Location Relation | 1 | 48 | 0.02 | .903 | .00 |
| Animal Identity Relation | 1 | 48 | 1.68 | .201 | .03 |
| Response Relation x Object-centered Location Relation | 1 | 48 | 38.60 | .000 | .45 |
| Response Relation x World-centered Location Relation | 1 | 48 | 49.28 | .000 | .51 |
| Object-centered Location Relation x World-centered Location Relation | 1 | 48 | 1.40 | .243 | .03 |
| Response Relation x Animal Identity Relation | 1 | 48 | 5.81 | .020 | .11 |
| Object-centered Location Relation x Animal Identity Relation | 1 | 48 | 0.27 | .606 | .01 |
| World-centered Location Relation x Animal Identity Relation | 1 | 48 | 2.23 | .142 | .04 |
| Response Relation x Object-centered Location Relation x World-centered Location Relation | 1 | 48 | 0.49 | .486 | .01 |
| Response Relation x Object-centered Location Relation x Animal Identity Relation | 1 | 48 | 0.03 | .869 | .00 |
| Response Relation x World-centered Location Relation x Animal Identity Relation | 1 | 48 | 0.01 | .907 | .00 |
| Object-centered Location Relation x World-centered Location Relation x Animal Identity Relation | 1 | 48 | 0.46 | .499 | .01 |
| Response Relation x Object-centered Location Relation x World-centered Location Relation x Animal Identity Relation | 1 | 48 | 0.02 | .893 | .00 |

*Note.* *df_Num_* indicates degrees of freedom numerator. *df_Den_* indicates degrees of freedom denominator. η^2^_p_ indicates partial eta-squared.
